# Heterometallic Li/Zn, Li/Al and Li/In catalysts for *rac*-lactide ring-opening polymerisation: “ate” or “non-ate” pathways?

**DOI:** 10.1039/d5cy00872g

**Published:** 2025-09-03

**Authors:** Thitirat Piyawongsiri, Anand J. Gaston, Maisarah Abdul Rahman, Jack W. J. Hughes, George E. Rudman, Phoebe A. Lowy, Gary S. Nichol, Carole A. Morrison, Khamphee Phomphrai, Jennifer A. Garden

**Affiliations:** a Department of Materials Science and Engineering, School of Molecular Science and Engineering, Vidyasirimedhi Institute of Science and Technology (VISTEC) Thailand khamphee.p@vistec.ac.th; b EaStCHEM School of Chemistry, University of Edinburgh EH9 3FJ UK j.garden@ed.ac.uk

## Abstract

The ring-opening polymerisation (ROP) of lactide (LA) is an attractive route to produce aliphatic polyesters, with bimetallic catalysts displaying some of the highest catalyst activities to date. While a range of heterometallic catalysts have been reported to outperform their homometallic analogues, the origins of cooperativity are not always well understood. Previous studies indicate that the reaction pathways may differ for different metal heterocombinations, especially when an alkali metal is combined with zinc or aluminium. Here, a series of homo- and hetero-metallic complexes combining Li with Al, Zn or In, supported by an asymmetric methyl-ester substituted salen ligand (**H**_**2**_**L**), have been synthesised and characterised by single-crystal X-ray diffraction, to probe potential differences. The heterobimetallic **LLiZnCl**, **LLiAlCl**_**2**_, and **LLiInCl**_**2**_ complexes were all active for *rac*-LA ROP in the presence of an epoxide initiator, with **LLiInCl**_**2**_ offering the most efficient polymerisation while homobimetallic **LLi**_**2**_ was inactive. Investigations into the roles of the different metals through X-ray diffraction and DFT structural studies suggest that oxophilicity, Lewis acidity, and electronegativity difference between the two metals all play a role, with the high oxophilicity and Lewis acidity of Al overriding the “ate” pathway.

## Abstract 2

Heterometallic cooperativity can improve catalyst performance in lactide polymerisation, yet some heterocombinations do not cooperate. This work shows how the metal roles change with different heterocombinations, affecting polymerisation efficiency.

## Graphical abstract

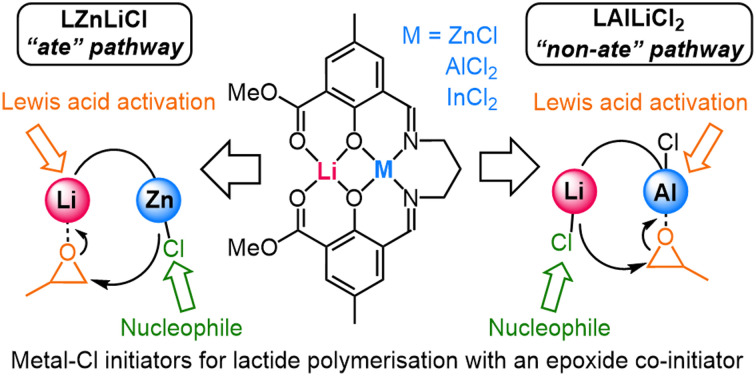

## Introduction

Bioderived and biodegradable poly(lactic acid) (PLA) is an attractive material for decreasing society's dependence on petroleum-based polymers. Typically prepared through the ring-opening polymerisation (ROP) of lactide (LA), PLA has wide-ranging uses including packaging and biomedical applications.^1–5^ While most metal-based catalyst development has focused on monometallic catalysts, harnessing heterometallic (mixed-metal) cooperativity has emerged as a promising method of enhancing catalyst performance. Heterobimetallic catalysts can improve polymerisation activity through multiple methods, including *via* multisite interactions and/or through the formation of “ate” complexes.^6–9^

**Fig. 1:**
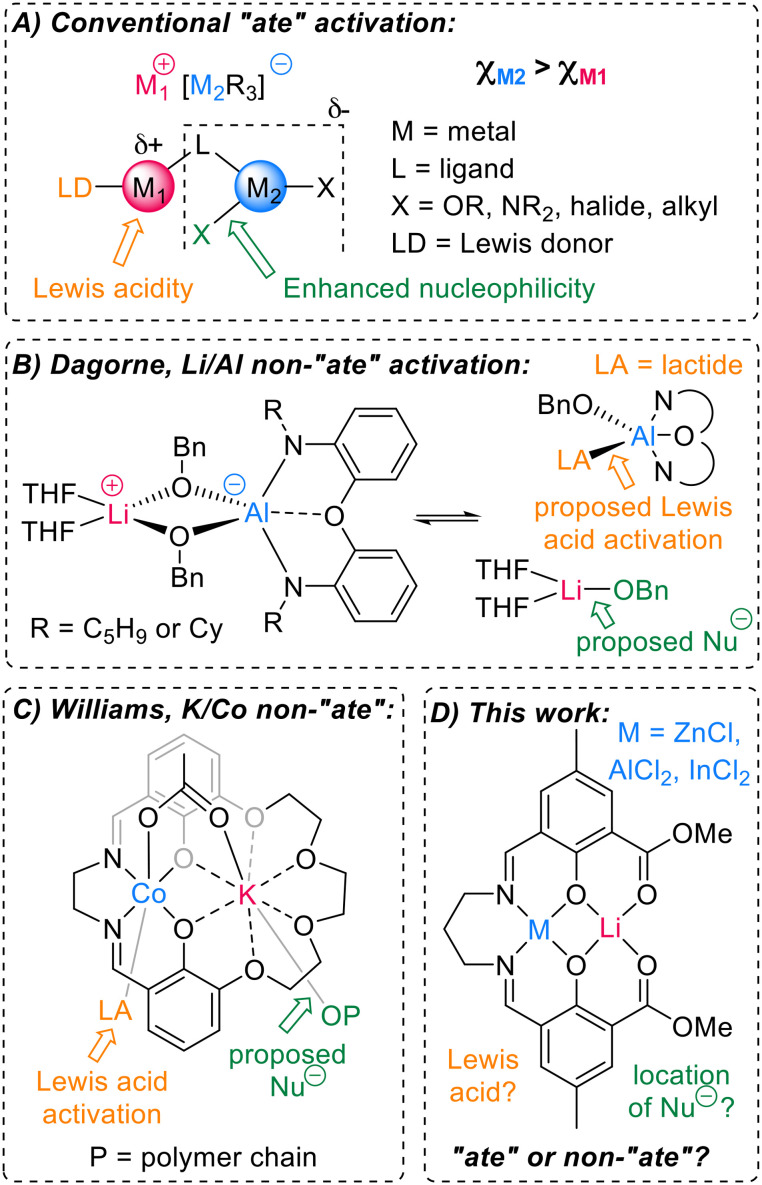
Examples of literature reported heterometallic complexes including A) general structure of lower-order alkali metal “ate” complexes;^25^ B) heterometallic Li/Al initiator for LA ROP reported by Dagorne *et al.*, proposed to operate through a non-“ate” pathway;^14^ C) heterometallic K/Co(iii) ROP catalyst reported for LA ROP by Williams *et al.*, proposed to operate *via* a non-“ate” pathway;^13^ D) heterometallic Li/Zn, Li/Al and Li/In explored for LA ROP in this work. Refer to Fig. S31 for in the SI for additional literature catalysts reported for ROP and ROCOP, where an alkali metal is paired with Zn, Al or In.

Coined by Wittig, the term “ate” is used to indicate the anionic formulation of a metal centre. For example, trisphenyl lithium zincate (LiZnPh_3_) displays a distinct chemistry to either of the homometallic components due to the anionic activation of Zn by the surrounding Ph anions.^10^ In a heterobimetallic environment, the softer, more carbophilic metal possesses an anionic formulation *e.g.* [ZnPh_3_]^−^, as the negative charge of the carbanions lies predominantly towards the metal with the highest electronegativity (Fig. 1A, *χ*_Zn_ = 1.65 > *χ*_Li_ = 0.98).^11^ “Ate” complexes can also be formed when two metals are connected by a bridging unit (*e.g.* alkoxide, halide, amide), creating an electronic communication between the two metals (Fig. 1A).^6,12^ Most of the cooperative heterometallic catalysts reported for cyclic ester ROP feature “ate” structures, consisting of a hard metal (*e.g.* alkali metal) combined with a softer metal (*e.g.* Zn, Al, In), connected by an alkoxide unit (see Fig. S31 for examples).^13–22^ In both small molecule and macromolecular transformations, the “ate” character can simultaneously enhance the Lewis acidity of one metal (M_1_) and the nucleophilicity of a M_2_–X group (*e.g.* X = alkyl in alkylation reactions or alkoxide initiating group/propagating polymer chain in ROP, Fig. 1A).^23^ Studies on heterometallic complexes supported by the Trost ProPhenol ligand showed that a greater electronegativity difference between two heterometals gave higher catalyst activities (K/Zn_2_ > Na/Zn_2_ > Ca/Zn > Mg/Zn).^20,23,24^ Yet this is not always the case. In particular, heterometallic alkali metal (AM)/aluminium combinations are not always cooperative in LA ROP, with several AM/Al systems displaying poorer activity than their homometallic counterparts.^14,16,21,22^

Harnessing the combined benefits of AM initiators (which typically show outstanding activity) with aluminium salen complexes (which often deliver highly controlled tacticity) is an attractive target. In 2010, Dagorne and co-workers reported lithium aluminate complexes that successfully polymerised LA at ambient temperature (Fig. 1B, left), whereas the homometallic components did not.^14^ During polymerisation, the LA monomer was proposed to coordinate to the Lewis acidic Al with LiOBn providing the source of the nucleophile (Fig. 1B, right). This mirrors insights from the field of epoxide/anhydride ring-opening copolymerisation (ROCOP), where experimental and computational studies on a series of AM/Al catalysts showed that Al coordinates the epoxide monomer and the AM provides the carboxylate nucleophile in the rate-determining step.^19^ These studies indicate that AM/Al catalysts do not necessarily follow “ate” activation pathways in ROP or ROCOP.

Studies on related Na/Co(iii), K/Co(iii) and alkaline earth metal (AEM)/Co(iii) catalysts showed a similar trend for LA ROP, where Co(iii) coordinates the Lewis basic monomer^13,26^ with the AM/AEM providing the alkoxide nucleophile (Fig. 1C). To gain understanding into the relative Lewis acidities of different metals, Kumar and Blakemore used ^31^P NMR studies to show a correlation between Lewis acidity and the p*K*_a_ value of the corresponding [metal(OH_2_)_*n*_]^*m*+^ complexes; the higher the p*K*_a_ value, the lower the Lewis acidity of the metal.^27^ As the p*K*_a_ values of many [metal(OH_2_)_*n*_]^*m*+^ complexes have been documented,^28^ this provides a facile and highly useful method of assigning the relative Lewis acidities of two different metals.^27^ Williams and co-workers elegantly translated this concept to LA ROP (as well as epoxide/anhydride and epoxide/CO_2_ ROCOP), showing that in the aforementioned AM/Co(iii) and AEM/Co(iii) complexes (Fig. 1C), less Lewis acidic AM/AEM gave higher catalyst activities due to more labile and thus more nucleophilic AM/AEM-alkoxide bonds (Fig. 1C).^26^ The Lewis acidity also gives a useful indication of the preferred monomer coordination site. Notably, the p*K*_a_ values of [Al(iii)(OH_2_)]^3+^ and [Co(iii)(OH_2_)]^3+^ are both relatively low, indicating that these metals are highly Lewis acidic (p*K*_a_ = 13.8 for Li(OH_2_)_*n*_^+^, *versus* 5.0 for Al(OH_2_)_*n*_^3+^ and 0.7 for Co(OH_2_)_*n*_^3+^; lower p*K*_a_ correlates to higher Lewis acidity). This may contribute to the preferential LA coordination at Co or Al compared to the alkali metal in these cases (Fig. 1B and C).

Given that several studies indicate that AM/Al catalysts do not necessarily follow an “ate” pathway (*e.g.*Fig. 1B), we wondered: can the high Lewis acidity of Al override the formation of “ate” complexes in LA ROP? This may change the reaction pathway compared to catalysts based on other heterometallic combinations, such as lithium zincates. Zn and Al have very similar electronegativities (*χ*_Al_ = 1.61; *χ*_Zn_ = 1.65) yet markedly different p*K*_a_ values for the aqua complexes (5.0 for Al(OH_2_)_*n*_^3+^*versus* 9.0 for Zn(OH_2_)_*n*_^2+^).^28^ As Li complexes have shown good activity in LA ROP,^15,17,29–31^ we synthesised and characterised heterometallic Li/Zn and Li/Al complexes, to probe the potential for “ate” (zinc) and “non-ate” pathways (aluminium) within LA ROP (Fig. 1D). The reactivity of these complexes was also benchmarked against Li/In as the larger and less Lewis acidic analogue of Al, to understand how the choice of specific heterocombinations influences the role of each metal in LA ROP.

## Results and discussion

### Synthesis and characterisation

**Scheme 1:**
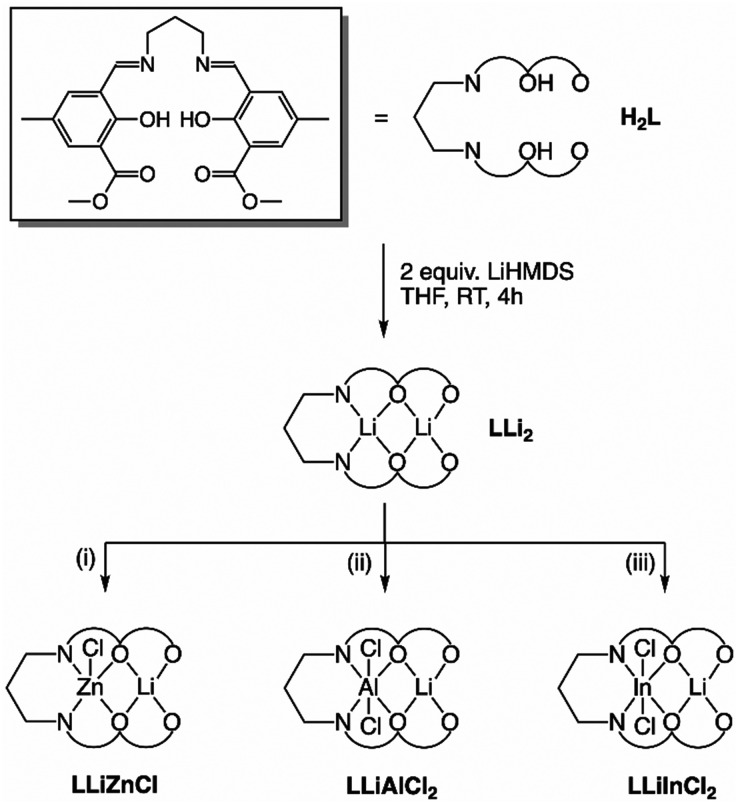
Formation of complexes **LLi**_**2**_, **LLiZnCl**, **LLiAlCl**_**2**_, and **LLiInCl**_**2**_ from **H**_**2**_**L**. Reaction conditions: (i) 1 equiv. ZnCl_2_ in THF at RT, 16 h; (ii) 1 equiv. AlCl_3_ in THF at RT, 16 h; (iii) 1 equiv. InCl_3_ in THF at RT, 16 h.

Most of the heterometallic Li complexes reported to date are based on symmetric ligand scaffolds, rather than asymmetric ligand scaffolds designed to hold two different metal centres in close proximity under polymerisation conditions. We previously reported a series of heterometallic Li/Al, Mg/Al, Ca/Al and Zn/Al catalysts based on an asymmetric *ortho*-carboxylic acid substituted salen ligand,^22^ with Al-chloride groups that ring-open an epoxide co-initiator to form a metal alkoxide for LA ROP *in situ* (Fig. S31).^32,33^ However, the Li/Al catalyst displayed poor activity, which was attributed to a ligand OH unit that participates in chain transfer reactions. The analogous methyl ester substituted ligand **H**_**2**_**L** was designed to overcome this challenge (Scheme 1). Importantly, **H**_**2**_**L** contains two distinct metal binding pockets (N_2_O_2_ and O_2_O_2_) for two different metals, yet it is dianionic instead of tetraanionic. This opens up access to heterocombinations of monovalent lithium with divalent (Zn) and trivalent (Al, In) metals whilst retaining at least one metal-chloride initiating unit. This propylene diamine backbone was selected because of the high activity compared to other diamine linkers reported in the literature.^33–35^

The methyl ester salen ligand **H**_**2**_**L** was therefore synthesised through the condensation of propylene diamine and the corresponding aldehyde (refer to SI for additional synthetic details). Homobimetallic **LLi**_**2**_ was synthesised by di-deprotonation of **H**_**2**_**L** with 2 equiv. of LiHMDS in THF at room temperature. The resultant bis-Li product **LLi**_**2**_ was characterised by X-ray diffraction studies as well as multinuclear NMR experiments (Fig. S5, S6 and S26). The molecular structure of **LLi**_**2**_ contains two independent dimers within the unit cell; the formation of a central Li_4_O_4_ cubane cluster in **LLi**_**2**_ is a typical aggregation structure for lithium alkoxides.^36,37^ Each Li is pentacoordinate and occupies a square pyramidal geometry, either coordinated to 3 phenolic oxygens and 2 carbonyl oxygens (O_2_O_2_ pocket, *τ*_5_ = 0.042)^38^ or coordinated to 3 phenolic oxygens and 2 imine nitrogens (N_2_O_2_ pocket, *τ*_5_ = 0.033).^38^ The *τ*_5_ value represents the geometry of penta-coordinate compounds; when *τ*_5_ is 0.00, the geometry is square pyramidal, whilst *τ*_5_ values close to 1.00 represent a trigonal bipyramidal geometry. Synthesis of the heavier alkali metal analogue Na was also investigated. However, reaction of **H**_**2**_**L** with two equivalents of NaH indicated that only one Na was incorporated into the ligand scaffold. In the ^1^H NMR spectrum, the ligand ^1^H NMR resonances were shifted compared to the **H**_**2**_**L** precursor, signifying deprotonation, and only one set of ligand resonances were observed (Fig. S13). Yet an OH resonance was present at 14.35 ppm with an integration of [1H] relative to the ligand resonances, confirming mono-deprotonation due to the larger ionic radius of Na disfavouring di-deprotonation. Indeed, Na and Ca are diagonal neighbours in the periodic table with similar ionic radii, and previous studies have shown that Ca tends to sit above the ligand plane in related salen(Ca) and aminophenol(Ca) complexes, which can prevent di-deprotonation.^18,22,39^ Therefore, bis-Li **LLi**_**2**_ was selected as the alkali metal precursor for subsequent transmetallation reactions.

**Fig. 2:**
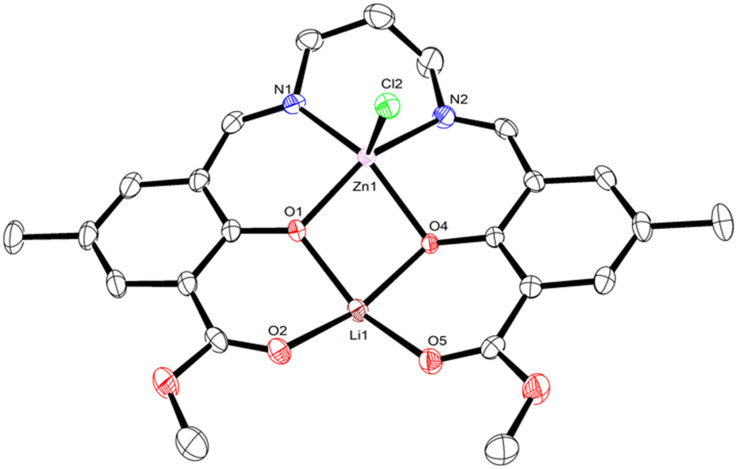
Monomeric component of the molecular structure of **LLiZnCl**, with displacement ellipsoids at the 50% probability level and hydrogen atoms removed for clarity. Selected bond lengths (Å) and angles (°): Zn1–Cl2 2.299(8), Zn1–O1 2.007(2), Zn1–O4 2.108(2), Zn1–N1 2.088(3), Zn1–N2 2.090(3), O1–Li1 1.982(6), O2–Li1 1.972(6), O4–Li1 2.001(6), O5–Li1 1.965(6), O1–Zn1–O4 79.2(8), O1–Zn1–N1 88.9(1), O1–Zn1–N2 151.0(1), N1–Zn1–O4 148.2(9), N1–Zn1–N2 92.8(1), N2–Zn1–O4 84.3(1), Li1–O1–Zn1 100.5(2), Li1–O4–Zn1 96.5(2), O1–Li1–O4 82.5(2), O2–Li1–O1 86.1(2), O2–Li1–O4 158.3(3), O5–Li1–O1 151.7(3), O5–Li1–O2 93.6(2), O5–Li1–O4 88.0(2). Zn1–Li1 distance 3.067(1) Å.

**Fig. 3:**
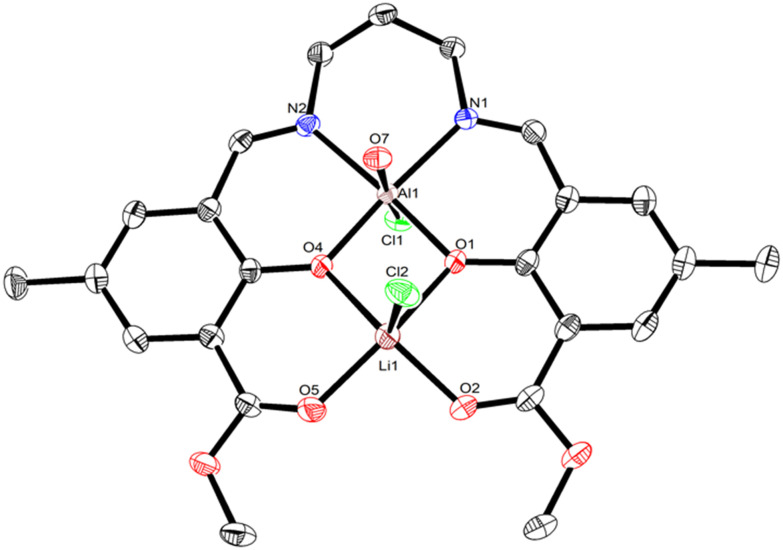
The molecular structure of **LLiAlCl**_**2**_**·H**_**2**_**O** with displacement ellipsoids at the 50% probability level and hydrogen atoms removed for clarity. Selected bond lengths (Å) and angles (°): Cl1–Al1 2.279(7), Cl2–Li1 2.508(4), Al1–O1 1.853(1), Al1–O4 1.862(1), Al1–N1 2.047(2), Al1–N2 2.023(2), O1–Li1 2.016(4), O2–Li1 1.901(4), O4–Li1 1.998(4), O5–Li1 1.905(4), O1–Al1–O4 83.2(6), O1–Al1–N1 90.9(6), O1–Al1–N2 173.0(7), O4–Al1–N1 171.8(7), O4–Al1–N2 91.5(6), N2–Al1–N1 94.0(7), Al1–O1–Li1 99.2(1), Al1–O4–Li1 99.5(1), O2–Li1–O1 88.1(2), O2–Li1–O4 145.2(2), O2–Li1–O5 92.8(2), O4–Li1–O1 75.8(1), O5–Li1–O1 156.2(2), O5–Li1–O4 90.4(2). Al1–Li1 distance 2.948(1) Å.

**Fig. 4:**
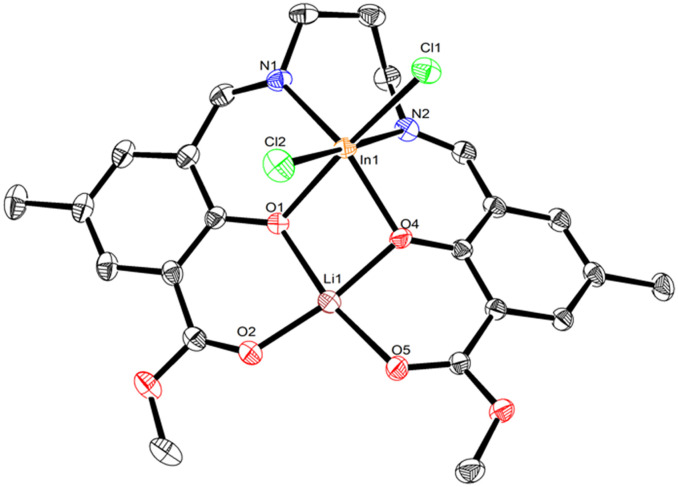
The molecular structure of **LLiInCl**_**2**_ with displacement ellipsoids at the 50% probability level and hydrogen atoms removed for clarity. Selected bond lengths (Å) and angles (°): In1–Cl1 2.448(4), In1–Cl2 2.465(5), In1–O1 2.189(1), In1–O4 2.116(1), In1–N1 2.207(2), In1–N2 2.306(2), O1–Li1 2.035(4),^38^ O2–Li1 1.882(4), O4–Li1 1.908(3), O5–Li1 2.003(4), O4–In1–O1 74.1(5), N1–In1–O1 83.5(5), N1–In1–O4 147.4(5), N2–In1–O1 100.0(5), N2–In1–O4 80.5(5), N2–In1–N1 80.6(6), Li1–O1–In1 93.6(1), Li1–O4–In1 99.7(1), O2–Li1–O1 88.0(1), O4–Li1–O1 82.2(1), O4 Li1–O2 170.2(2), O5–Li1–O1 168.4(2), O5–Li1–O2 103.6(2), O5–Li1–O4 86.2(1). In1–Li1 distance: 3.079(1) Å.

Subsequent addition of one equivalent of ZnCl_2_, AlCl_3_, or InCl_3_ to **LLi**_**2**_ in THF at room temperature followed by overnight stirring generated heterometallic **LLiZnCl**, **LLiAlCl**_**2**_, and **LLiInCl**_**2**_, respectively (Scheme 1). **LLiZnCl** was crystallised from DCM, while **LLiAlCl**_**2**_ and **LLiInCl**_**2**_ were crystallised by vapor diffusion in 1,2-dichloroethane/hexane. X-ray diffraction studies confirmed the heterometallic structures of these three complexes (Fig. 2–4). The molecular structure of heterometallic **LLiZnCl** contains two independent dimers within its asymmetric unit, which are essentially identical, and half of each dimer is formed by an inversion symmetry operation generating equivalent atoms.

Similar to homobimetallic **LLi**_**2**_, heterometallic **LLiZnCl** crystallises as a dimer bridging through the oxophilic lithium atoms (Fig. S26 and S27). The pentacoordinate zinc in **LLiZnCl** displays a low *τ*_5_ value (0.048 and 0.031)^38^ indicative of square-pyramidal geometry. Notably, the chloride is ligated to Zn, indicating the presence of a lithium zincate structure. The Zn–Cl initiating group in **LLiZnCl** is directed away from the bridging Li–O bonds, at least in the solid-state structure. Moreover, each Li coordinates to 3 phenolic oxygens and 2 carbonyl oxygens in a slightly distorted square pyramidal geometry (*τ*_5_ = 0.110 and 0.062).^38^ Rather than forming a cubane structure similar to bis-Li **LLi**_**2**_, heterometallic **LLiZnCl** forms an open ladder-type structure featuring Zn–O, O–Li, Li–O and O–Zn rungs.

Extending beyond monovalent Li or divalent Zn, complexes **LLiAlCl**_**2**_ and **LLiInCl**_**2**_ combine Li with a trivalent Al or In metal centre. X-ray diffraction studies showed that **LLiAlCl**_**2**_ crystallised with one equivalent of water present in the structure, which likely originated from trace water present during crystallisation. Both **LLiAlCl**_**2**_**·H**_**2**_**O** and **LLiInCl**_**2**_ are octahedral at Al and In, have monomeric structures in the solid state, and have two chloride co-ligands present to balance the charge. In all cases, the heterometal (Zn, Al, In) occupies the inner N_2_O_2_ pocket. The location of In, Al, Zn, and Li can be rationalised by the relative hard–soft acid–base interactions between the metals and oxygen/nitrogen in the asymmetric ligand structure. In **LLi**_**2**_, lithium and oxygen are small and hard ions, thus the Li–O bond would be expected to be stronger than a Li–N bond. Therefore, when a second, softer metal is added to **LLi**_**2**_, the weaker Li–N bond dissociates to coordinate the heterometal in the N_2_O_2_ ligand pocket. In all cases, only a single set of ligand structure resonances is observed in both the ^1^H and ^13^C NMR spectra, suggesting that the metal placement is selective. The NC*H*_2_^1^H NMR resonances of the propylene backbone in **LLiAlCl**_**2**_ and **LLiInCl**_**2**_ are shifted downfield compared to **LLi**_**2**_ (4.10–4.16 ppm for **LLiAlCl**_**2**_ and **LLiInCl**_**2**_*vs.* 3.19–3.21 ppm for **LLi**_**2**_, all in CDCl_3_, Fig. S5, S7 and S9). As well as a downfield shift of the NC*H*_2_^1^H NMR resonances of **LLiZnCl**, these are also split (3.68 and 4.27 ppm). The ester (O=*C*OCH_3_) ^13^C NMR resonance is essentially identical at approximately 169 ppm in CDCl_3_ for homometallic **LLi**_**2**_ and all three heterometallic complexes **LLiZnCl**, **LLiAlCl**_**2**_, and **LLiInCl**_**2**_, providing further evidence that Li is situated in the O_2_O_2_ pocket in all cases (Fig. S6, S8, S10 and S12). In contrast, the imine (N=*C*H) ^13^C NMR resonances of the heterometallic complexes are shifted in comparison to bis-Li (164.4 ppm for **LLi**_**2**_*vs.* 169.2–167.1 ppm for **LLiZnCl**, **LLiAlCl**_**2**_, and **LLiInCl**_**2**_, all in CDCl_3_, Fig. S6, S8, S10 and S12). This information provides further support that the metal placements are maintained in the solution state, with the heterometal (Zn, Al or In) occupying the inner N_2_O_2_ pocket.

Looking at the structure of **LLiAlCl**_**2**_ shows an intriguing difference to lithium zincate **LLiZnCl**. One equivalent of adventitious water is present in the structure of **LLiAlCl**_**2**_, and importantly, this water molecule coordinates to Al. This suggests that the higher Lewis acidity of Al *versus* Li favours coordination of a Lewis donor at aluminium (p*K*_a_ = 13.8 for Li(OH_2_)_*n*_^+^, 5.0 for Al(OH_2_)_*n*_^3+^; higher p*K*_a_ correlates to lower Lewis acidity). In the presence of water, one chloride is ligated to Li, whereas the second bonds to Al, indicating the lack of a lithium aluminate structure. This is a notable difference from the molecular structures of both **LLiZnCl** and **LLiInCl**_**2**_, where the co-ligand(s) bond to the more electronegative metal to form an “ate” structure (*χ*_Li_ = 0.98, *χ*_Al_ = 1.61; *χ*_Zn_ = 1.65, *χ*_In_ = 1.78). The ^1^H NMR spectrum of **LLiAlCl**_**2**_ in CDCl_3_ showed no resonance for water and indicated that the two chloride units coordinate to Al in a *trans* arrangement (Fig. S9), as no splitting was observed in the propylene diamine backbone region. This indicates the magnetic equivalence of the protons in the axial and equatorial positions of the six-membered Al–N–C*H*_2_–C*H*_2_–C*H*_2_–N ring.^40,41^ Taken together, these observations indicate that a Cl ion can migrate from Al to Li upon coordination of a Lewis base (*e.g.* cyclic ester monomers), supporting the notion that a Lewis donor can override the formation of a lithium aluminate.

The molecular structure of **LLiInCl**_**2**_ shows a lithium indate structure where the two chloride co-ligands are bound to In in a *cis* arrangement. The ^1^H NMR spectrum of **LLiInCl**_**2**_ shows broad peaks in the NC*H*_2_–C*H*_2_ region suggesting a rapid exchange of chloride atom between In and Li metals. However, we cannot rule out the possibility of the Ray–Dutt racemisation mechanism in an octahedral geometry, which could give similar NMR character.^42^ The ionic radii of Zn^2+^ (74 pm), Al^3+^ (53.5 pm), and In^3+^ (80 pm) also influence the metal–metal distance in the heterometallic complexes, resulting in Zn–Li, Al–Li, and In–Li distances of 3.067 Å, 2.948 Å, and 3.079 Å, respectively.^43^ Previous studies have shown that intermetallic proximity in the range of 3–5 Å can deliver improved catalyst performance in lactide polymerisation and CO_2_/epoxide ring-opening copolymerisation,^6,7^ and thus heterometallic **LLiZnCl**, **LLiAlCl**_**2**_ and **LLiInCl**_**2**_ were investigated for LA ROP.

### Ring-opening polymerisation of *rac*-lactide

**Scheme 2:**
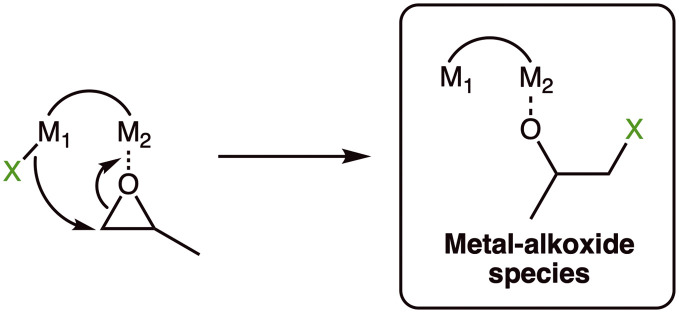
Proposed generation of an active metal-alkoxide species from a metal chloride precursor and epoxide co-initiator.

All four complexes were tested for their activity for *rac*-lactide (*rac*-LA) ROP. Catalysts, epoxide, and *rac*-LA were each mixed in a 1 : 50 : 100 ratio in toluene. Polymerisations carried out at room temperature were unsuccessful due to the low solubility of the catalyst. Therefore, the polymerisations were conducted at 120 °C in line with the conditions from previous literature for Al–Cl initiators. Previous studies also showed that using a 1 : 1 catalyst : epoxide ratio significantly decreased catalyst activity, which was likely due to inefficient initiation.^22^ While metal-chloride units are often not capable of efficiently initiating LA ROP, metal-chlorides have been shown to ring-open an epoxide instead to generate an active metal-alkoxide species *in situ* (Scheme 2).^32–34,44–47^

The most common epoxide, propylene oxide (PO), was used to initiate *rac*-LA ROP producing the desired PLA (Table 1, entries 1–4). Catalysts **LLiZnCl**, **LLiAlCl**_**2**_, and **LLiInCl**_**2**_ displayed activity towards LA ROP without epoxide homopolymerisation (Fig. S25, refer to SI for further details), while bis-Li complex **LLi**_**2**_ was inactive due to the lack of a metal-chloride initiating group. Indeed, our previous studies have shown that Li-half salen complexes displayed low activities due to the lack of a labile Li-alkoxide unit, although the addition of benzyl alcohol (BnOH) as external initiator boosted the activity by switching the polymerisation to an activated monomer mechanistic pathway.^31^

**Table 1:** Ring-opening polymerisation of *rac*-lactide (*rac*-LA) with a propylene oxide (PO) or cyclohexene oxide (CHO) co-initiator^a^

Entry	Catalyst	Initiator	Time (min)	% conv.^b^	*M*_n,cal_ c (kDa)	*M*_n,obs_ d (kDa)	*Đ* d
1	**LLi**_**2**_	PO	120	0	—	—	—
2	**LLiZnCl**	PO	180	60	8.6	3.5	1.37
3	**LLiAlCl**_**2**_	PO	240	73	5.3	2.9	1.47
4	**LLiInCl**_**2**_	PO	30	74	5.4	6.4	3.19
5	**LLiInCl**_**2**_	CHO	20	73	5.3	5.0	1.62

a Reaction condition: [catalyst]/[epoxide]/[*rac*-LA] = 1 : 50 : 100, [*rac*-LA] = 1 M in toluene, 120 °C.

b Determined by ^1^H NMR spectroscopy.

c Calculated as ([*rac*-LA]/[catalyst] × (% conversion/100) × MW of lactide)/no. of initiator (one initiator for **LLiZnCl** and two initiators for **LLiAlCl**_**2**_ and **LLiInCl**_**2**_).

d Determined by size-exclusion chromatography (SEC) in THF, refractive index (RI) detector relative to polystyrene standard, *M*_n_ was calculated using the correction factor of *M*_n,obs_ = 0.58 × *M*_n,SEC_.

Of the four complexes tested, **LLiInCl**_**2**_ showed the fastest activity for *rac*-LA ROP, converting 74 equivalents within 30 min whereas **LLiZnCl** and **LLiAlCl**_**2**_ took up to 8 times longer to reach similar conversions (Table 1). However, **LLiInCl**_**2**_ gives a notably poor control over the polymer dispersity (*Đ* = 3.19). To stay below the boiling point of the epoxide and target improved dispersities, cyclohexene oxide (CHO) was used instead of PO (b.p PO = 34 °C, b.p CHO = 130 °C). The use of CHO gave slightly faster polymerisation and significantly narrower dispersity (Table 1, entries 4 *versus* 5). This was attributed to the bulkier CHO reducing side reactions that can occur during polymerisation, such as chain transfer and transesterification.^45^ All active catalysts gave no tacticity control, forming atactic PLA with *P*_i_ values ranging from 0.45 to 0.48 (Fig. S22–S24).

**Fig. 5:**
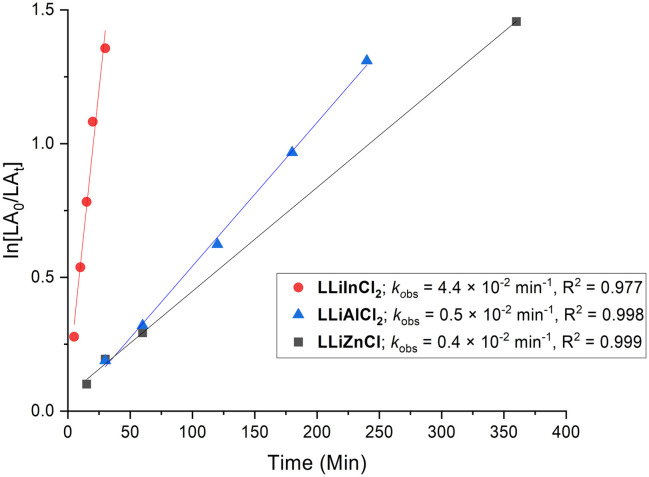
Kinetic plot of ln[LA_0_/LA_*t*_] *versus* time for the ROP of *rac*-LA catalysed by **LLiZnCl**, **LLiAlCl**_**2**_ and **LLiInCl**_**2**_. Reaction conditions: [catalyst]/[PO]/[*rac*-LA] = 1 : 50 : 100, [*rac*-LA] = 1 M in toluene, and 120 °C.

Kinetic studies were also performed for all catalysts (Fig. 5). The polymerisations were all first order in monomer and proceeded without an induction period. The relative rates of polymerisation were determined as **LLiInCl**_**2**_ (*k*_obs_ = 4.4 × 10^−2^ min^−1^) ≫ **LLiAlCl**_**2**_ (*k*_obs_ = 0.5 × 10^−2^ min^−1^) > **LLiZnCl** (*k*_obs_ = 0.4 × 10^−2^ min^−1^). These studies show that the nature of the heterometal has a significant effect on the polymerisation activity beyond the different number of initiating groups. Specifically, **LLiInCl**_**2**_ and **LLiAlCl**_**2**_ both have two chloride units, yet the *k*_obs_ value of **LLiInCl**_**2**_ is almost ten times greater than that of **LLiAlCl**_**2**_. Overall, the kinetic studies suggest that the catalyst activity trend of **LLiInCl**_**2**_ ≫ **LLiAlCl**_**2**_ > **LLiZnCl** is thus linked to the number of metal-chloride initiating units, combined with the trends in metal ionic radii (In^3+^ > Zn^2+^ > Al^3+^) and electronegativity difference (Δ*χ*_InLi_ = 0.80 > Δ*χ*_ZnLi_ = 0.67 > Δ*χ*_AlLi_ = 0.63).^11^ Notably, **LLiAlCl**_**2**_ displays higher activity than several other Li/Al catalysts reported for LA ROP, including the *ortho*-carboxylic acid substituted salen Li/Al analogue, a Li/Al alkoxide catalyst based on a NON diamido ligand, and a Li/Al phenolate complex (see Fig. S31 for details).^14,16,22^ However, it is important to note that different reaction conditions were used, which limits these comparisons. In contrast, the activity of **LLiZnCl** and **LLiInCl**_**2**_ are both lower than other Li/Zn and Li/In complexes reported in literature, including Li/Zn complexes supported by a bisphenol ligand^17^ and a heterometallic Li/In dialkoxy-diimino salen ligand (Fig. S31).^15^

MALDI-TOF end-group analysis revealed the presence of cyclic PLA oligomers with all three heterometallic catalysts (Fig. S18–S21), suggesting that intramolecular transesterification (ring-closure) occurs. Catalysts **LLiAlCl**_**2**_ and **LLiInCl**_**2**_ show a mixture of cyclic and linear PLA, while **LLiZnCl** shows only cyclic PLA. It is worth noting that cyclic PLA would give the same MALDI-TOF peaks as a linear PLA polymer initiated by an enolate formed *via* lactide deprotonation, as reported previously.^48^ However, linear PLA features a distinctive HOC*H*– end group ^1^H NMR resonance (*δ* = 4.35 ppm) which was negligible in the cyclic polymers produced here, providing further evidence for cyclic polymer formation. Transesterification has been observed previously with some other heterometallic catalysts, and was attributed to the metal–metal′ proximity and the formation of “ate” structures.^24^ Specifically, the coordination of a polymer ester unit to a Lewis acidic metal centre facilitates transesterification, with a proximal metal-alkoxide unit potentially providing the nucleophile (Fig. S32). It is worth noting that cyclic PLA oligomers tend to show better ionisation in MALDI-TOF analysis, and thus the cyclic species in the mass spectra may be overestimated, as MALDI-TOF is a non-quantitative method for polymer analysis.^49^ The major series in the polymer samples catalysed by **LLiInCl**_**2**_ features the chloro-propylene oxide or chloro-cyclohexene oxide end groups (Fig. S20 and S21), confirming that the metal-chloride acts as an initiator for epoxide ring-opening followed by insertion of lactide in agreement with previous reports.^22,32,44,45,50^ While catalyst **LLiAlCl**_**2**_ shows chloro-propylene oxide end groups, it also shows an oligomeric series featuring hydroxyl-terminated PLA.

### Simulation of coordination sites

**Fig. 6:**
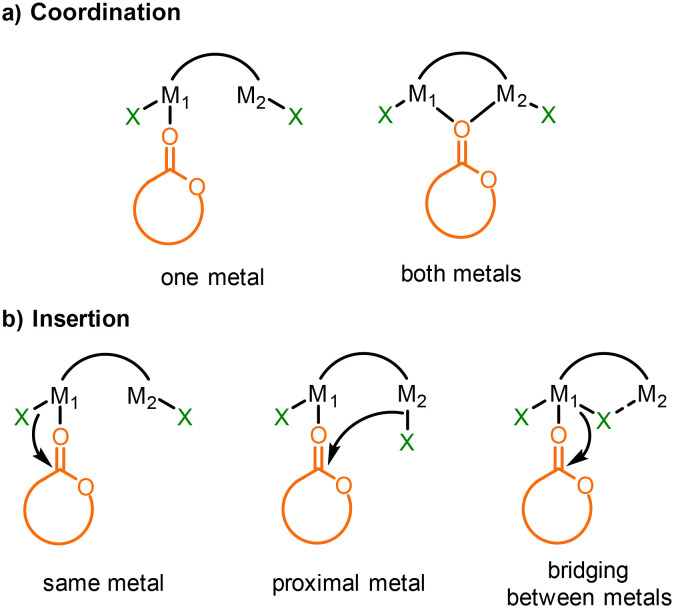
Potential mechanisms for the initiation of lactide ring-opening polymerisation (where X is a nucleophile such as an alkoxide or a propagating polymer chain).

Most metal-based catalysts for LA ROP follow a coordination–insertion mechanism,^6,26,51^ where the proposed rate determining step is often nucleophilic attack from a metal-alkoxide intermediate (M–OR, OR = propagation polymer chain) upon a metal-coordinated lactide monomer. In the case of multimetallic or heterometallic catalysts, the pathways are more complicated, as the monomer can coordinate to either, or both metals (Fig. 6, top).^7^ Similarly, nucleophilic attack from an initiator can occur from the same metal that coordinates the monomer, from the nearby heterometal, or even from a bridging group (Fig. 6, bottom). Here, the activity trend (In > Al > Zn) does not directly correlate with the Lewis acidity (based on p*K*_a_ values of the corresponding aqua complexes, Lewis acidity of Al > In > Zn), or the electronegativity difference between the two metals (Δ*χ*_InLi_ = 0.80 > Δ*χ*_ZnLi_ = 0.67 > Δ*χ*_AlLi_ = 0.63)^11^ or indeed the oxophilicity (*θ*_Al_ = 0.8 > *θ*_In_ = 0.4 > *θ*_Li_ = 0.3 > *θ*_Zn_ = 0.2).^52^ This indicates that other factors are also at play. The lower number of initiating units of **LLiZnCl** is likely to be a key contributor to the lower activity of this catalyst. The catalysts may also operate through different mechanisms.

**Fig. 7:**
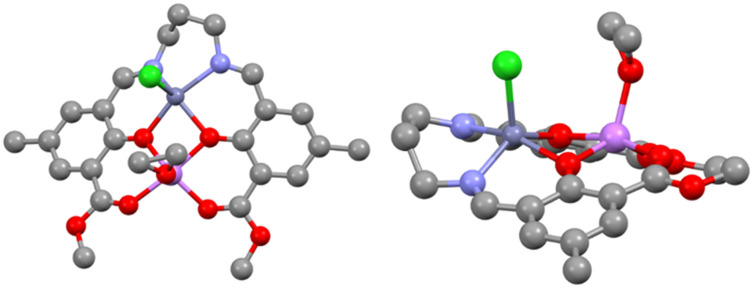
Top (left) and side (right) views of geometry optimised complex **LLiZnCl** of the more stable configurations upon epoxide coordination. Colour scheme: grey = C, light purple = N, red = O, dark purple = Zn, green = Cl, pink = Li. H atoms omitted for image clarity.

**Scheme 3:**
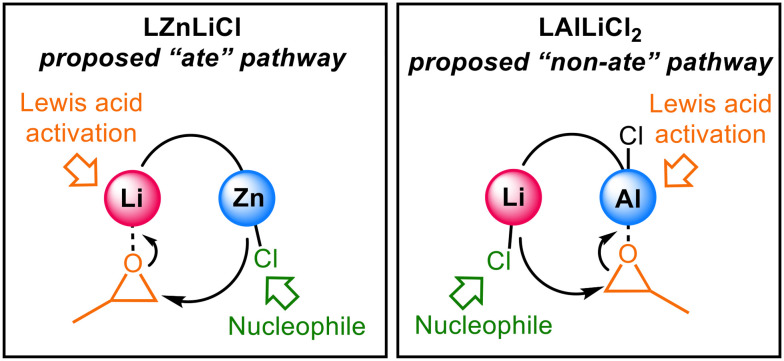
Proposed initiation mechanism *via* “ate” (Li/Zn) and “non-ate” (Li/Al) pathways.

The molecular structure of **LLiAlCl**_**2**_**·H**_**2**_**O** shows that a Lewis base can preferentially coordinate at Al, causing migration of a chloro-group to Li and overriding the formation of a lithium aluminate structure. In terms of LA ROP, this observation is consistent with previous literature reports (*vide supra*, Fig. 1B),^13,14^ indicating that **LLiAlCl**_**2**_ is likely to not operate *via* an “ate” pathway, with monomer coordination instead occurring at Al and with Li providing the source of a nucleophile. We were curious to understand which metal would favour epoxide coordination in **LLiZnCl**, and whether the lithium zincate structure would be maintained in the presence of an epoxide. Geometry optimisation calculations were therefore performed for **LLiZnCl** at the B3LYP/6-311G* level to determine the site of epoxide coordination (Fig. 7, see SI for additional details). Density-functional theory (DFT) simulations support the coordination of the epoxide to lithium in **LLiZnCl**, providing support for **LLiZnCl** initiating LA ROP *via* an “ate” intermediate. Furthermore, the calculations show that epoxide coordination preferentially occurs on the same face as the Zn–Cl unit of **LLiZnCl** (Table S10). This positions the Zn–Cl initiating unit in close proximity with the epoxide, facilitating intramolecular epoxide opening by the Zn–Cl unit. The DFT simulations also show that the Zn–Cl bond length of **LLiZnCl** increases from 2.303 to 2.330 Å upon epoxide coordination (Table S11), weakening the Zn–Cl bond and thus labilising the chloride unit towards nucleophilic attack and ring-opening of the epoxide. This is further supported by the local force constant for the Zn–Cl bond, which is reduced from 0.958 mdyn Å^−1^ to 0.851 mdyn Å^−1^ upon epoxide binding (Table S11).^49^ These observations suggest a key mechanistic difference between the Li/Zn and Li/Al catalysts, and indicate that Lewis base coordination occurs preferentially at Al > Li > Zn. This does not exactly correlate with the p*K*_a_ values of the metal (aqua) complexes (p*K*_a_, Li > Zn > Al, which suggests Lewis acidity Al > Zn > Li). However, the observed trend matches well with the oxophilicities of the metals, with aluminium being the most oxophilic and zinc the least (*θ*_Al_ = 0.8 > *θ*_In_ = 0.4 > *θ*_Li_ = 0.3 > *θ*_Zn_ = 0.2).^52^ Therefore, O-donor Lewis base coordination occurs at Al over Li, yet the lower oxophilicity of Zn switches the monomer coordination site to Li, maintaining an “ate” structure (Scheme 3).

With **LLiInCl**_**2**_, the DFT studies show that epoxide binding preferentially occurs at Li, maintaining an “ate” structure (Table S12, Fig. S30). This is attributed to the significantly larger electronegativity difference between the two metals (Δ*χ*_InLi_ = 0.80 > Δ*χ*_ZnLi_ = 0.67 > Δ*χ*_AlLi_ = 0.63).^11^ Similar to **LLiZnCl**, epoxide coordination occurs on the same face as the In–Cl bond, although the preference is more slight (by Δ*G* = 4.0 kJ mol^−1^ for **LLiInCl**_**2**_ and by 13.5 kJ mol^−1^ for **LLiZnCl**). Notably, the local force constants of **LLiInCl**_**2**_ show significant differences between the In binding to the two “halves” of the salen ligand; one set of In–N, In–O and In–Cl bonds are significantly stronger than the other (Table S14) due to the “butterfly wing” folding of the ligand in **LLiInCl**_**2**_ (Fig. 4). Upon epoxide coordination, one of the In–Cl bonds becomes weaker, with a lower local force constant of 1.078 mdyn Å^−1^ compared to 1.147 mdyn Å^−1^. In contrast, the other In–Cl bond becomes stronger, with an increase in the local force constant from 0.78 mdyn Å^−1^ to 1.037 mdyn Å^−1^. Taken together, these observations not only show the usefulness of local force constant calculations as a method of probing bond strength for metal-chloride initiators, but also demonstrate that electronic communication through the metal–O–metal′ scaffold means that epoxide coordination at one metal centre modulates the metal′-chloride bond at the other metal′ centre. Overall, this shows that there are multiple factors which determine whether the ROP of LA follows an “ate” pathway or not.

## Conclusions

A series of bimetallic complexes derived from a methyl ester salen ligand scaffold were synthesised, characterised and tested for the ROP of *rac*-LA. The molecular structures **LLiAlCl**_**2**_ and **LLiInCl**_**2**_ were monomeric, while **LLi**_**2**_ and **LLiZnCl** displayed dimer structures in the solid state. Both **LLiAlCl**_**2**_ and **LLiInCl**_**2**_ require two chloride co-ligands to balance the chelated metal charges, creating more steric hindrance that can prevent the metal centres from approaching each other closely enough to form a dimer. The location of In, Al, Zn, and Li can be rationalised by the relative hard–soft acid–base interactions between the metals and oxygen/nitrogen in the asymmetric ligand structure. The heterobimetallic catalysts were all active for *rac*-LA ROP with a catalyst activity order of **LLiInCl**_**2**_ ≫ **LLiAlCl**_**2**_ > **LLiZnCl**, while homobimetallic **LLi**_**2**_ was inactive. The coordination sites, electronegativity differences and metal Lewis acidity all affect the catalytic activity. The role of each metal in initiation *via* epoxide coordination and ring-opening was probed, to understand the differences between these heterometallic catalysts. The molecular structure of **LLiZnCl** and **LLiAlCl**_**2**_, combined with DFT studies, indicate that the initiation modes are different for these two complexes, with **LLiZnCl** operating *via* an “ate” pathway and **LLiAlCl**_**2**_ operating *via* a Lewis acid pathway. Notably, the site of the epoxide coordination appears to be determined by the metal oxophilicity and Lewis acidity, with the epoxide coordinating to the Al metal centre in **LLiAlCl**_**2**_, but to Li in **LLiZnCl** and **LLiInCl**_**2**_, which preserves the formation of the lithium zincate and lithium indate structures. These studies indicate that there is a complex interplay between the metal Lewis acidity, electronegativity and oxophilicity in governing the structures formed and thus polymerisation pathways. This study flags the importance of fundamental understanding into how different heterometallic combinations may govern the polymerisation mechanism, and some of the levers that can be pulled to design next generation heterometallic catalysts for LA ROP, and beyond.

## Author contributions

J. A. Garden and K. Phomphrai: conceptualisation, supervision, writing and editing. T. Piyawongsiri, A. J. Gaston, G. E. Rudman, and P. A. Lowy: synthesis, characterisation, and writing. J. W. J. Hughes: mass spectrometry characterisation of polymers. G. S. Nichol: single crystal X-ray analyses and the refinement of the structures. M. A. Rahman and C. A. Morrison: computational calculations. All authors have given approval to the final version of the manuscript.

## Conflicts of interest

There are no conflicts to declare.

## Supplementary Material

CY-015-D5CY00872G-s001

CY-015-D5CY00872G-s002

## Data Availability

Supplementary information: Synthesis details, characterisation data for complexes and polymers including NMR spectroscopy, mass spectrometry, and single crystal X-ray diffraction data are included in the SI. See DOI: https://doi.org/10.1039/D5CY00872G. CCDC 2445764 (for **LLi**_**2**_), 2445763 (for **LLiZnCl**), 2445761 (for **LLiAlCl**_**2**_) and 2445762 (for **LLiInCl**_**2**_) contain the supplementary crystallographic data for this paper.^53*a*–*d*^
