# Supplementary material for: Heterometallic Li/Zn, Li/Al and Li/In catalysts for rac-lactide ring-opening polymerisation: “ate” or “non-ate” pathways?
Source: Catal Sci Technol. 2025 Sep 3;15(20):6113–21. doi: 10.1039/d5cy00872g (PMC12407087; doi:10.1039/d5cy00872g)
Supplement: CY-015-D5CY00872G-s001 [file CY-015-D5CY00872G-s001.pdf]

Supplementary Information (SI) for Catalysis Science & Technology.
This journal is © The Royal Society of Chemistry 2025

Electronic Supporting Information

Heterometallic Li/Zn, Li/Al and Li/In catalysts for *rac*-lactide ring-opening polymerisation: "ate" or "non-ate" pathways?

Thitirat Piyawongsiri [a], Anand J. Gaston [b], Maisarah Abdul Rahman [b], Jack W. J. Hughes [b], George E. Rudman[b], Phoebe A. Lowy [b], Gary S. Nichol [b], Carole A. Morrison[b], Khamphee Phomphrai [*a], Jennifer. A. Garden [*b]

[a] Department of Materials Science and Engineering, School of Molecular Science and Engineering, Vidyasirimedhi Institute of Science and Technology (VISTEC), Thailand
E-mail: khamphee.p@vistec.ac.th

[b]EaStCHEM School of Chemistry, The University of Edinburgh, UK
Email: j.garden@ed.ac.uk

Table of Contents

Experimental Section *3*

Materials 3

Measurements 3

X-ray Crystallography 3

Synthesis of ligand and complexes *4*

General procedure for the ring opening polymerisation of rac-lactide *6*

NMR spectroscopy data *7*

SEC data from rac-LA ROP *14*

MALDI-ToF mass spectrometry of PLA *16*

Example calculation of PLA tacticity *18*

Example of purified PLA *20*

X-ray crystallography data *21*

Computational methods *28*

Overview of heterometallic systems reported in literature *30*

The features of heterometallic "ate" catalysts *30*

Experimental Section

Materials

All manipulations involving air- or moisture-sensitive compounds were performed in a glovebox and using standard Schlenk techniques under an argon atmosphere. All dry solvent was collected from a solvent purification system (Innovative Technologies), dried over activated 4 Å molecular sieves and stored under argon. Chloroform-*d* ($CDCl_3$) for NMR spectroscopy studies was degassed by three freeze−pump−thaw cycles and stored over activated 4 Å molecular sieves under argon atmosphere. *Rac*-lactide (*rac*-LA) was purified by double recrystallization in toluene followed by sublimation and stored in the glovebox freezer (-35 °C). Other reagents and solvents were obtained from Sigma-Aldrich, Fisher Scientific, Honeywell and Fluorochem and used without further purification. Methyl 5-methylsalicylate (**P0**) was synthesized according to the literature procedure.[1]

Measurements

All NMR spectra were recorded on a Bruker AVA500 spectrometer at 303 K and referenced to the protio impurity of commercial chloroform-*d* ($CDCl_3$, δ 7.26 ppm for ^{1}H NMR and 77.16 ppm for ^{13}C NMR) as an internal standard. Mass spectrometry analysis for **LLiAlCl₂** and **LLiInCl₂** complexes were carried out using Bruker Daltonics 12T SolariX Fourier Transform Ion Cyclotron Resonance mass spectrometer using atmospheric pressure photoionization (APPI). Mass analysis for pre-ligand **P1** (see page 4 for structure), **H_2L**, **LLi_2**, **LLiZnCl**, and polymers were carried out using MALDI mass spectrometry, collected on a Bruker Daltonics UltrafleXtreme™ MALDI-ToF/ToF MS instrument in reflectron mode. MALDI-ToF samples were made up fresh on the day of analysis in a 2:2:1 volume ratio of (a) polymer (10 mg mL^{-1}), dithranol (25 mg mL^{-1}) and NaTFA (10 mg mL^{-1}) in THF or (b) polymer in table 1, entry 2 (10 mg mL^{-1}), 2,5-dihydroxybenzoic acid (25 mg mL^{-1}) and NaTFA (10 mg mL^{-1}) in THF. A droplet (2 μL) of the resultant mixture was spotted on to the sample plate and submitted for MALDI-ToF MS analysis. The M_n of the MALDI-ToF polymer samples were analysed within a window of 1 -5 kDa. Elemental analyses were performed by Elemental Microanalysis Ltd. ICP-OES analysis was carried out on a Perkin Elmer Optima 5300DC Inductively Coupled Plasma Optical Emission Spectrometer. Samples were dissolved in 3%v/v nitric acid and submitted for ICP-OES analysis. SEC analyses of the filtered polymer samples were carried out in GPC grade THF at a flow rate of 1 mL min^{-1} at 35 °C on a 1260 Infinity II GPC/SEC single detection system with mixed bed C PLgel columns (300 ×7.5 mm).

X-ray Crystallography

Suitable crystals were selected and mounted on a MITIGEN holder in Paratone oil on a Rigaku Oxford Diffraction SuperNova diffractometer. The crystal was kept at a steady T = 120.00(10) K during data collection. The crystal structure was solved by a dual method using intrinsic phasing (SHELXT program)[2] and refined by full-matrix least squares against F^2 using the program SHELXL based on the ShelXle engine or Olex2 software package.[3] All non-H atoms were refined anisotropically, while the H atoms were placed in calculated positions and not refined. The crystallographic images were processed by the Ortep3 program.

Synthesis of ligand and complexes

Two different synthetic routes were investigated for the synthesis of **H_2L**. **Route 1** involved the formylation of 5-methylsalicylic acid *via* a Duff reaction to produce 3-formyl-5-methylsalicylic acid followed by a methyl esterification to prepare methyl 3-formyl-2-hydroxy-5-methylbenzoate (**P1**), and finally a one-pot Schiff base condensation with propylenediamine to obtain methoxy ester salen ligand (**H_2L**). **Route 2** involved the methyl esterification of 5-methylsalicylic acid into methyl 5-methylsalicylate (**P0**) followed by formylation *via* a Duff reaction to obtain **P1**. The final Schiff base condensation step from **P1** to **H_2L** was the same in both synthetic routes. **Route 2** was carried out as an alternative to **Route 1**, which overcome issues with low yields and reproducibility.

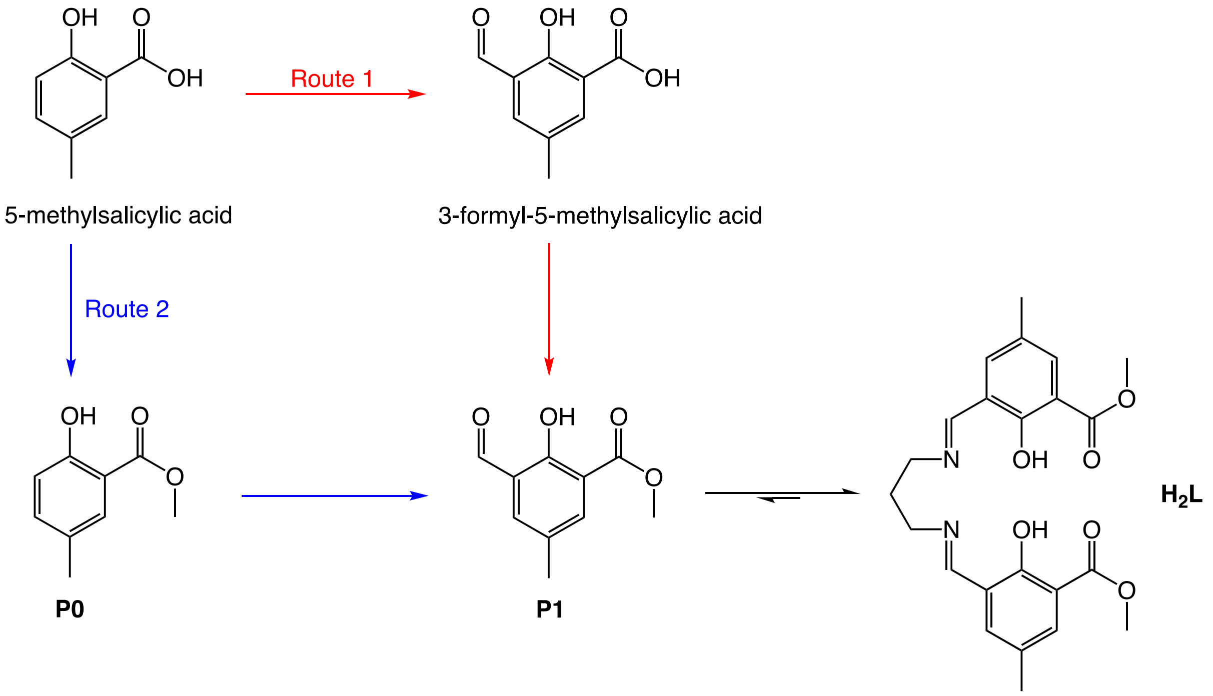

Scheme 1. Synthetic routes towards **H_2L** from 5-methylsalicylic acid staring material

Pre-ligand (P1). Hexamethylenetetramine (5.00 g, 36.0 mmol) was added to a solution of methyl 5-methylsalicylate (1.90 g, 12.0 mmol) in trifluoroacetic acid (25.0 ml). The solution was refluxed for 16 hours before hot 1 M HCl (75.0 ml) was added. After a further 16 hours of stirring, **P1** was isolated, washed and dried under vacuum to give an off-white solid in good yield (1.55 g, 8.00 mmol, 66%)

P1: ^{1}H NMR (500 MHz, $CDCl_3$, 303K) δ: 11.29 (s, 1H, O*H*), 10.46 (s, 1H, O=C*H*), 7.90 (dd, *J* = 2.5, 0.8 Hz, 1H, Ar-*H*), 7.81 (d, *J* = 2.4 Hz, 1H, Ar-*H*), 3.97 (s, 3H, O-C*H*$_3$), 2.32 (s, 3H, Ar-C*H*$_3$). ^{13}C NMR (126 MHz, $CDCl_3$) δ 189.41 (O=*C*H), 169.96 (O=*C*OCH_3) 161.95 (Ar*C*-OH), 136.87, 135.12 (Ar*C*-H), 128.68 (Ar*C*-CH_3), 124.14, 113.99 (Ar*C*-C, 52.75 (O-*C*H_3), 20.33 (Ar-*C*H_3). MALDI-ToF-MS: m/z [M + Na] $^{+}$: calculated 217.048; found 217.113.

Methyl ester salen ligand (H_2L). A solution of propylenediamine (0.11 g, 1.50 mmol) in ethanol was added dropwise to a solution of **P1** (0.58 g, 3.00 mmol) in ethanol, which upon reflux for 3 hours. After cooling for 16h, yellow needles were deposited from solution. These crystals were filtered and washed with cold ethanol (0.38 g, 0.89 mmol, 59%).

H_2L: ^{1}H NMR (500 MHz, $CDCl_3$, 303K) δ: 14.07 (s, 2H, O*H*), 8.41 (s, 2H, N=C*H*), 7.75 (dd, *J* = 2.3, 0.8 Hz, 2H, Ar-*H*), 7.33 – 7.30 (m, 2H, Ar-*H*), 3.94 (s, 6H, O-C*H*$_3$), 3.73 (td, *J* = 6.6, 1.3 Hz, 4H, N-C*H*$_2$), 2.31 (d, *J* = 0.8 Hz, 6H, Ar-C*H*$_3$), 2.16 – 2.07 (m, 2H, CH_2-C*H*$_2$). ^{13}C NMR (126 MHz, $CDCl_3$) δ 167.28 (O=*C*OCH$_3$), 164.09 (N=*C*H), 159.95 (Ar*C*-OH), 136.06, 135.16 (Ar*C*-H), 127.27 (Ar*C*-CH$_3$), 120.27 (Ar*C*-C=N), 117.62 (Ar*C*-COO), 56.91 (N-*C*H$_2$), 52.26 (O-*C*H$_3$), 31.55 (CH$_2$-*C*H$_2$), 20.31 (Ar-*C*H$_3$). MALDI-ToF-MS: m/z [M + Na]$^+$: calculated 449.169; found 449.259.

Methyl ester salen LLi_2 complex. Under argon atmosphere at RT °C, a solution of LiHMDS (0.04 g, 0.23 mmol) was added dropwise to a solution of **H_2L** (0.05 g, 0.12 mmol) in THF and stirred for 4 hours to from a pale-yellow solution. The solvent was removed *in vacuo*, resulting in the formation of an off-white powder. Recrystallisation in DCM yielded **1** (0.04 mg, 0.10 mmol, 82%) as colourless crystals.

Complex **LLi_2**: ^{1}H NMR (500 MHz, $CDCl_3$, 303K): δ = 7.90 (s, 2H, N=C*H*), 7.65 (d, J = 2.6 Hz, 2H, Ar-*H*), 7.05 (d, J = 2.7 Hz, 2H, Ar-*H*), 3.61 (s, 6H, O-C*H*$_3$), 3.22 – 3.17 (m, 4H, N-C*H*$_2$), 2.21 (s, 6H, Ar-C*H*$_3$), 1.49 (s, 2H, CH$_2$-C*H*$_2$). ^{13}C NMR (126 MHz, $CDCl_3$): δ = 169.4 (O=*C*OCH$_3$), 169.3 (Ar*C*-O), 164.4 (N=*C*H), 141.6, 135.1 (Ar*C*-H), 126.2 (Ar*C*-CH$_3$), 119.3 (Ar*C*-C=N), 118.6 (Ar*C*-COO), 66.1 (N-*C*H$_2$), 51.3 (O-*C*H$_3$), 32.5 (CH$_2$-*C*H$_2$), 20.3 (Ar-*C*H$_3$). MALDI-ToF-MS: m/z [M-2H]$^+$: calculated 438.20; found 438.27. Elemental Analysis: predicted (**1**·0.25 DCM): C 60.50%, H 5.79%, N 6.07%; found: C 60.94%, H 5.42%, N 6.06%.

Methyl ester salen LLiZnCl, $LLiAlCl_2$, and $LLiInCl_2$ complex. Under argon atmosphere at RT °C, MCl_x ($ZnCl_2$ = 0.01 g, $AlCl_3$ = 0.01 g, $InCl_3$ = 0.02 g, 0.11 mmol) was dissolved in the minimum volume of THF and added dropwise to complex **LLi_2** (0.05 g, 0.11 mmol) in THF, resulting in the formation of a yellow solution. The solution was stirred overnight. Afterwards, the resulting solution was filtered using 1,2-dichloroethane and transferred to a new Schlenk flask. The product was isolated by vacuum. Recrystallisation in DCM yielded **LLiZnCl** (0.04 g, 0.07 mmol, 62%) as pale green crystals. Recrystallisation in 1,2-dichloroethane/hexane by vapor diffusion yielded **$LLiAlCl_2$** (0.02 mg, 0.05 mmol, 45%) and **$LLiInCl_2$** (0.05 mg, 0.08 mmol, 70%) as colourless crystals.

Complex **LLiZnCl**: ^{1}H NMR (500 MHz, $CDCl_3$, 303K): δ = 8.09 (s, 2H, N=C*H*), 7.86 (d, J = 2.7 Hz, 2H, Ar-*H*), 7.13 (d, J = 2.6 Hz, 2H, Ar-*H*), 4.27 (t, J = 12.0 Hz, 2H, N-C*H*$_2$), 3.92 (s, 6H, O-C*H*$_3$), 3.67 (dd, J = 13.7, 6.2 Hz, 2H, N-C*H*$_2$), 2.37 – 2.26 (m, 1H, CH$_2$-C*H*$_2$), 2.23 (s, 6H, Ar-C*H*$_3$), 2.01 – 1.94 (m, 1H, CH$_2$-C*H*$_2$). ^{13}C NMR (126 MHz, $CDCl_3$): δ = 169.8 (O=*C*OCH$_3$), 169.2 (Ar*C*-O), 169.0 (N=*C*H), 142.7, 137.8 (Ar*C*-H), 122.1 (Ar*C*-CH$_3$), 121.4 (Ar*C*-C=N), 117.6 (Ar*C*-COO), 63.5 (N-*C*H$_2$), 51.9 (O-*C*H$_3$), 30.1 (CH$_2$-*C*H$_2$), 20.1 (Ar-*C*H$_3$). MALDI-ToF-MS: m/z [M-Cl]$^+$: calculated 495.11; found 495.14. Elemental Analysis: predicted (**LLiZnCl** •0.5 DCM): C 49.12%, H 4.38%, N 4.87%; found: C 48.83%, H 4.24%, N 4.72%.

Complex **LLiAlCl$_2$**: ^{1}H NMR (500 MHz, $CDCl_3$, 303K) δ 7.99 – 7.95 (m, 4H, N=C*H,* Ar-*H*), 7.27 (s, *J* = 2.5 Hz, 2H, Ar-*H*), 4.13 (t, *J* = 5.2 Hz, 4H, N-C*H*$_2$), 3.93 (s, 6H, O-C*H*$_3$), 2.42 – 2.34 (m, 2H, CH_2-C*H*$_2$), 2.27 (s, 6H, Ar-C*H*$_3$). ^{13}C NMR (126 MHz, $CDCl_3$) δ 169.13 (O=*C*OCH$_3$), 167.10 (N=*C*H), 162.75 (Ar*C*-O), 141.75, 138.84 (Ar*C*-H), 125.69 (Ar*C*-CH$_3$), 121.42 (Ar*C*-C=N), 117.09 (Ar*C*-COO), 61.98 (N-*C*H$_2$), 52.37 (O-*C*H$_3$), 26.92 (CH_2-*C*H$_2$), 20.08 (Ar-*C*H$_3$). APPI-MS: m/z [M-Cl] $^+$: calculated 493.1293; found 493.1324. The 1:1 Li:Al ratio was verified by ICP-OES analysis.

Complex **LLiInCl$_2$**: ^{1}H NMR (500 MHz, $CDCl_3$, 303K) δ 7.96 (d, *J* = 11.0 Hz, 4H, N=C*H,* Ar-*H*), 7.21 (d, *J* = 2.6 Hz, 2H, Ar-*H*), 4.16 (s, 4H, N-C*H*$_2$), 3.92 (s, 6H, O-C*H*$_3$), 2.56 (s, 2H, CH_2-C*H*$_2$), 2.25 (s, 6H, Ar-C*H*$_3$). ^{13}C NMR (126 MHz, $CDCl_3$) δ 169.22 (N=*C*H, O=*C*OCH$_3$), 167.43 (Ar*C*-O), 144.42, 139.07 (Ar*C*-H), 124.65 (Ar*C*-CH$_3$), 120.54, 119.23 (Ar*C*-C=N, Ar*C*-COO), 60.56 (N-*C*H$_2$), 52.34 (O-*C*H$_3$), 29.86 (CH_2-*C*H$_2$), 20.10 (Ar-*C*H$_3$). APPI-MS: m/z [M-Cl] $^+$: calculated 581.0516; found 581.0575. The 1:1 Li:In ratio was verified by ICP-OES analysis.

General procedure for the ring opening polymerisation of *rac*-lactide

The following representative procedure is for entry 1 in Table 1. In the glove box under an argon atmosphere, 100 equiv. of *rac*-lactide (0.20 g, 1.39 mmol), 1 equiv. of **LLi$_2$** (0.006 g, 0.01 mmol) and toluene (1M) were added into an air-tight Supelco™ glass vial equipped with a magnetic stirrer bar. 50 equiv. of propylene oxide (0.04 g, 0.70 mmol) were added (Supelco™ vials can withstand some pressure differential; note that the reactions were performed above the boiling point of propylene oxide). The vial was sealed tightly and heated to 120 °C using a DrySyn heating block whilst stirring. After the appropriate reaction time, the reaction was then removed from the heating block, cooled to room temperature and quenched with chloroform. The solvent was then removed using compressed air, the product dissolved in $CDCl_3$ and subsequently analysed using ^{1}H NMR spectroscopy.

NMR spectroscopy data

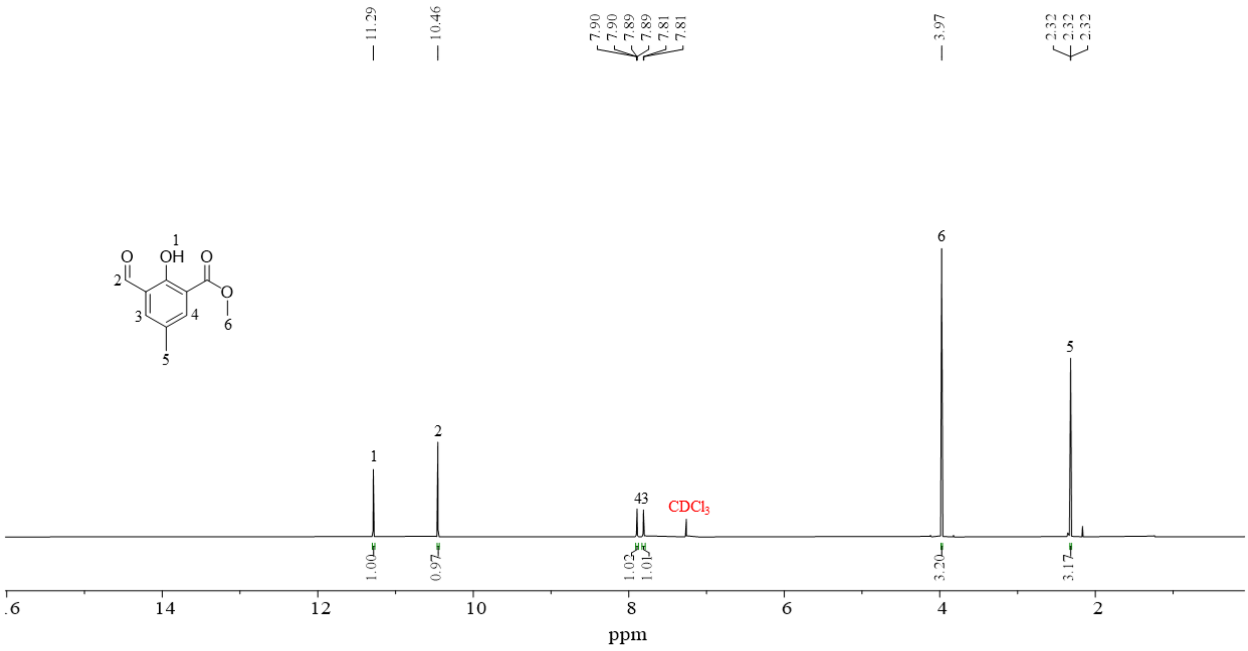

Figure S1. ^{1}H NMR spectrum (500 MHz, $CDCl_3$, 303K) of ligand precursor **P1**.

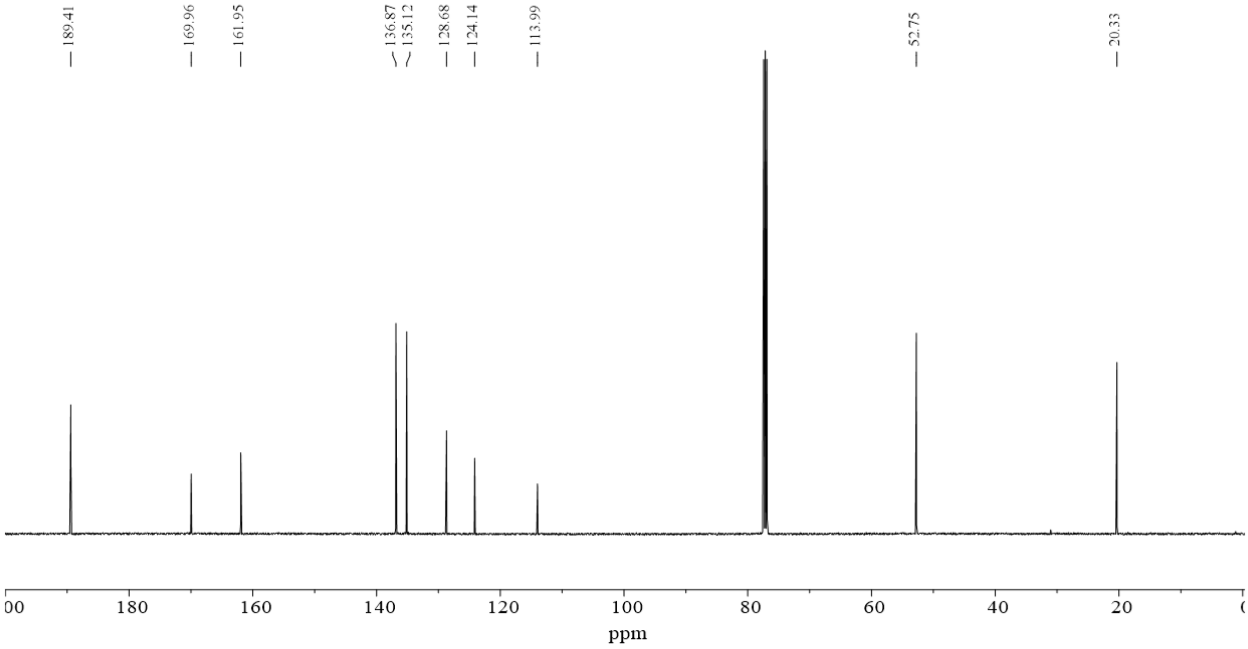

Figure S2. ^{13}C NMR spectrum (126 MHz, $CDCl_3$, 303K) of ligand precursor **P1.**

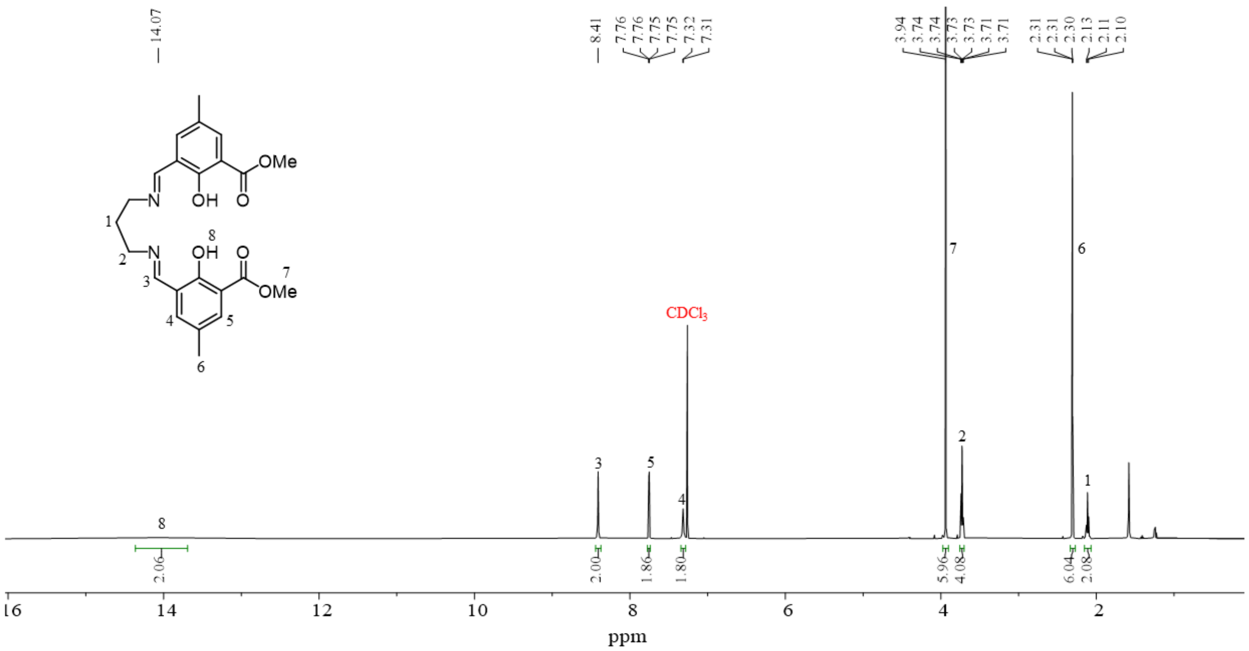

Figure S3. ^{1}H NMR spectrum (500 MHz, $CDCl_3$, 303K) of **H_2L**.

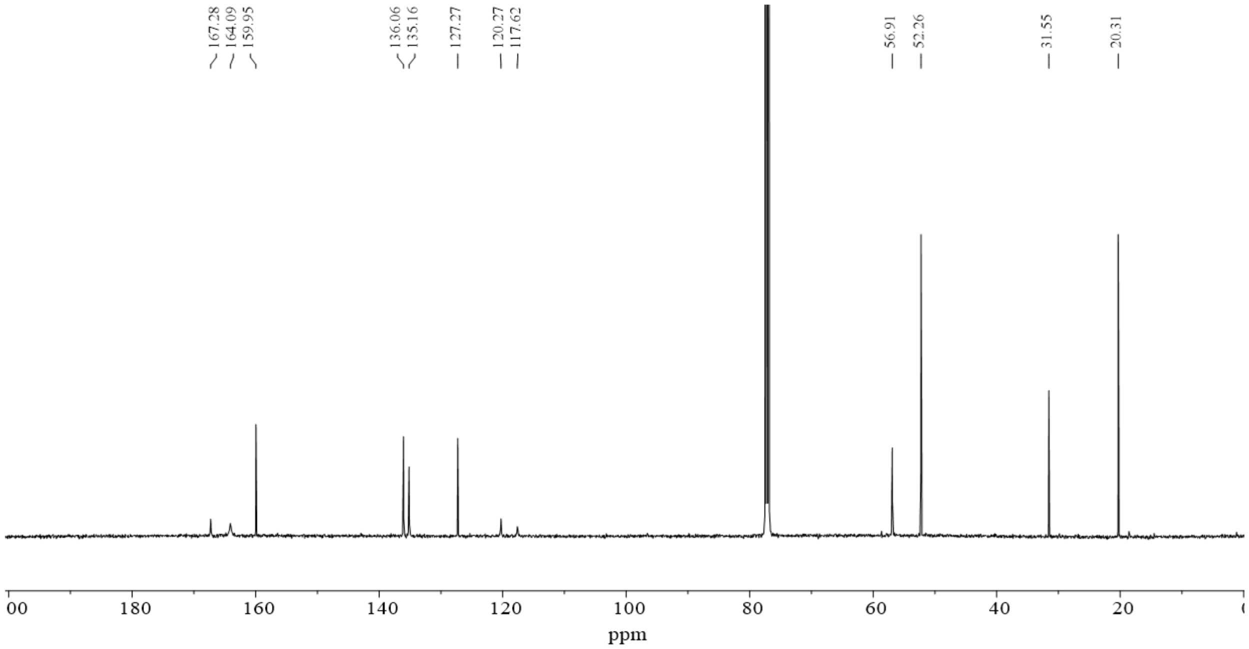

Figure S4. ^{13}C NMR spectrum (126 MHz, $CDCl_3$, 303K) of **H_2L**.

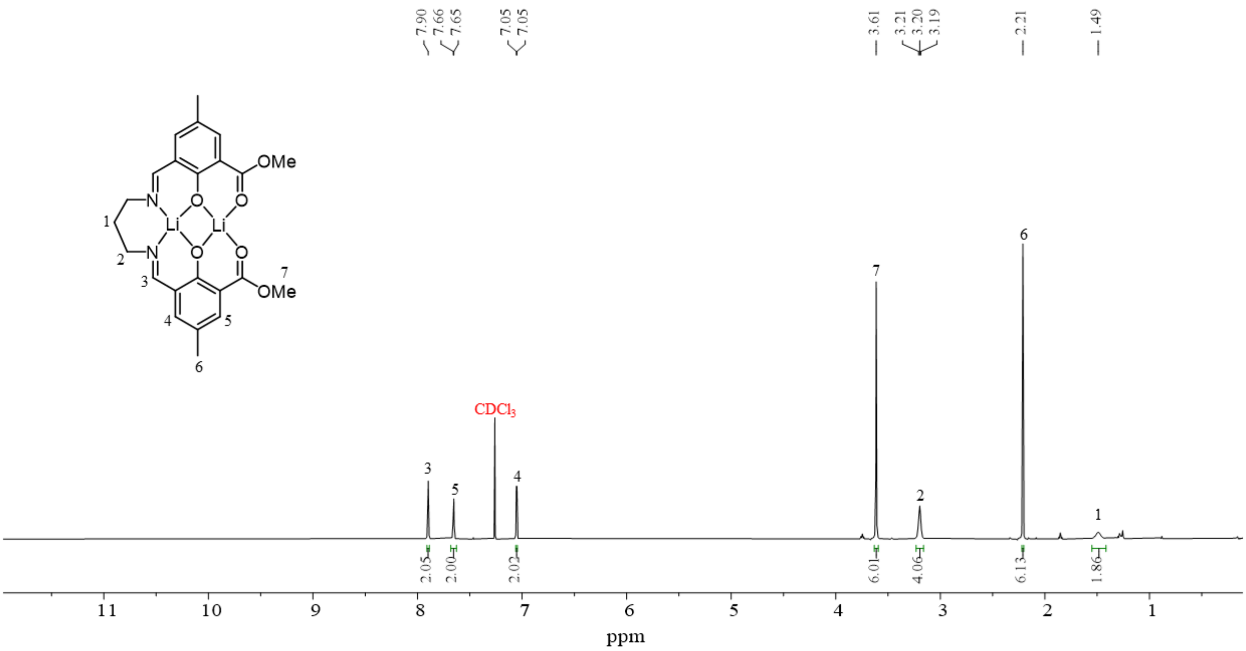

Figure S5. ^{1}H NMR spectrum (500 MHz, $CDCl_3$, 303K) of **LLi_2**.

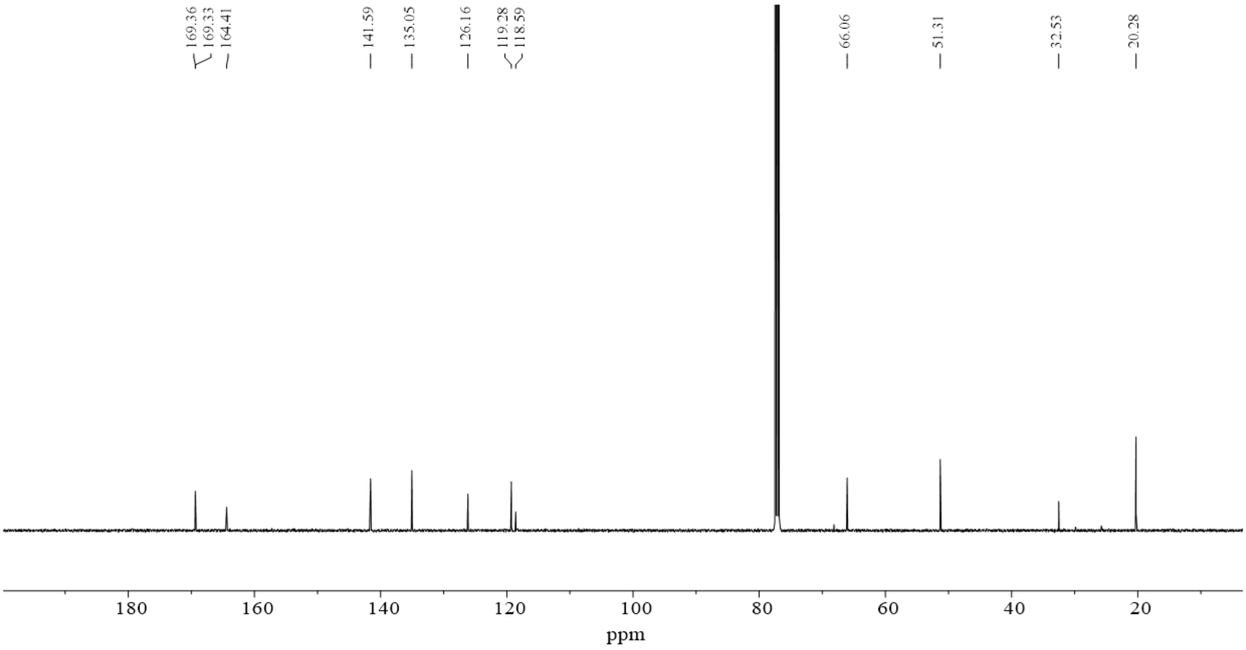

Figure S6. ^{13}C NMR spectrum (126 MHz, $CDCl_3$, 303K) of **LLi_2**.

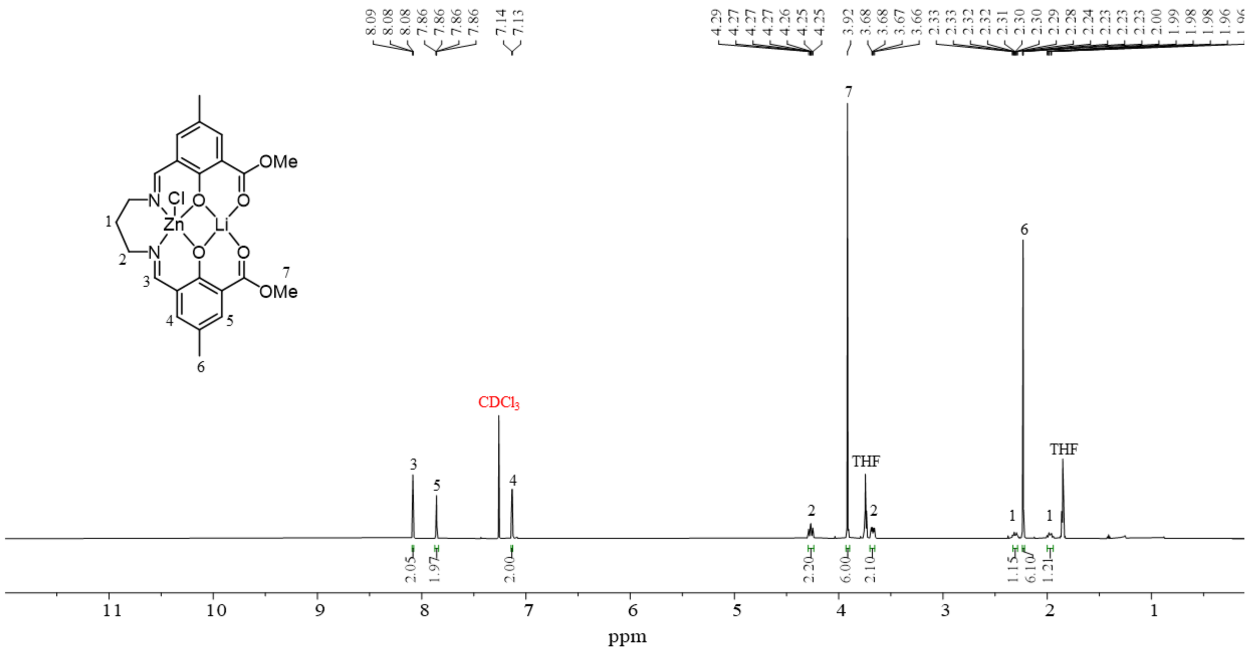

Figure S7. ^{1}H NMR spectrum (500 MHz, $CDCl_3$, 303K) of **LLiZnCl**.

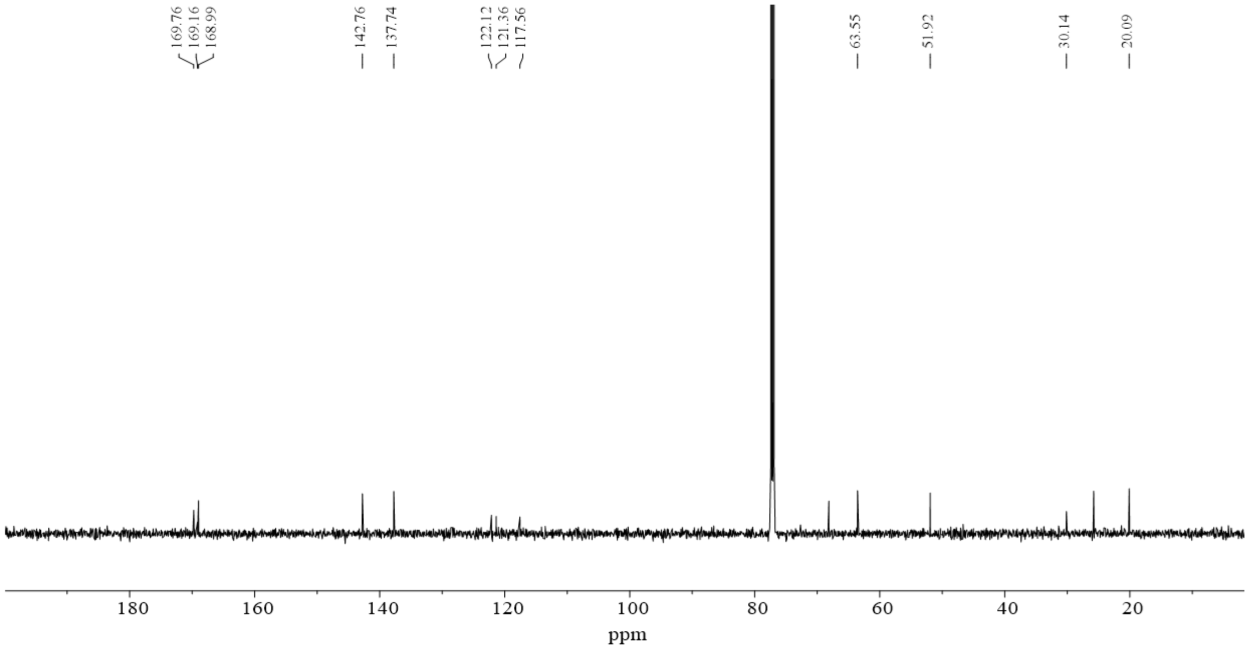

Figure S8. ^{13}C NMR spectrum (126 MHz, $CDCl_3$, 303K) of **LLiZnCl.**

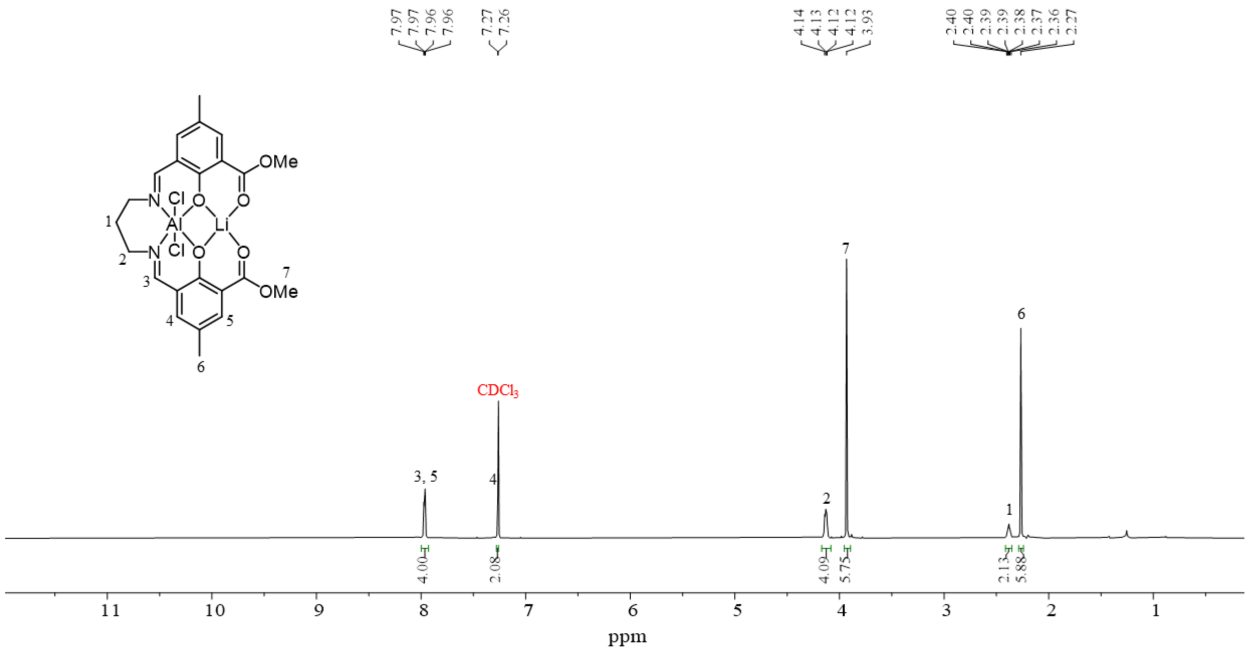

Figure S9. ^{1}H NMR spectrum (500 MHz, $CDCl_3$, 303K) of **$LLiAlCl_2$**.

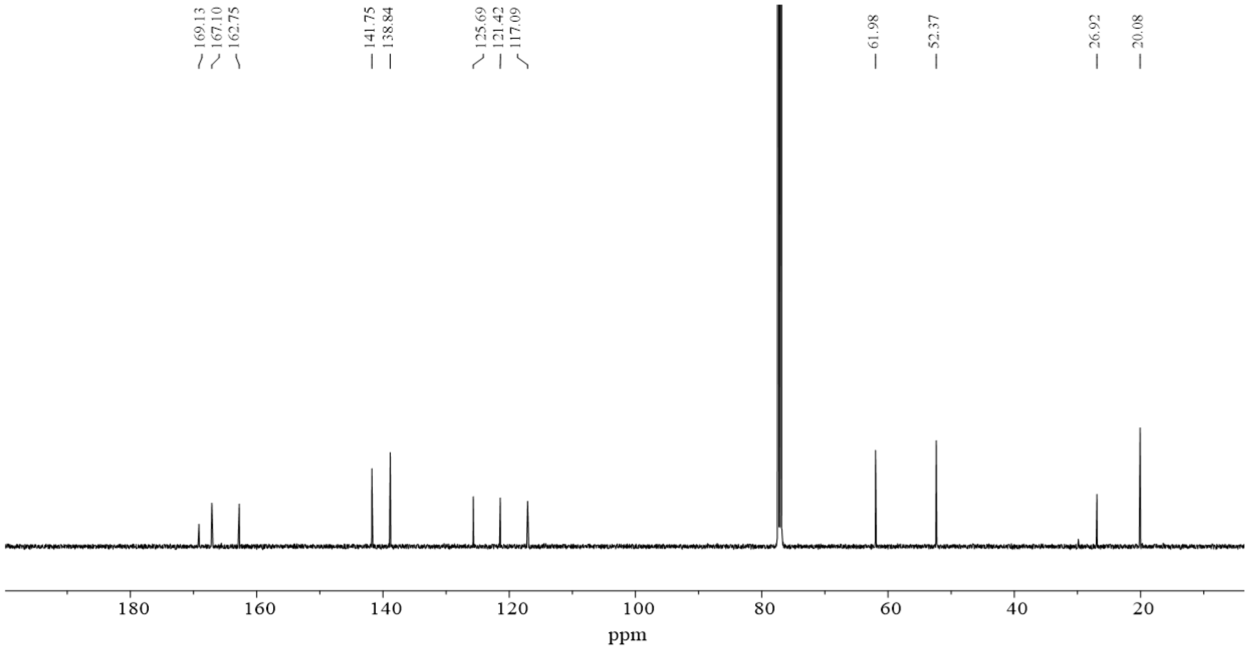

Figure S10. ^{13}C NMR spectrum (126 MHz, $CDCl_3$, 303K) of **$LLiAlCl_2$.**

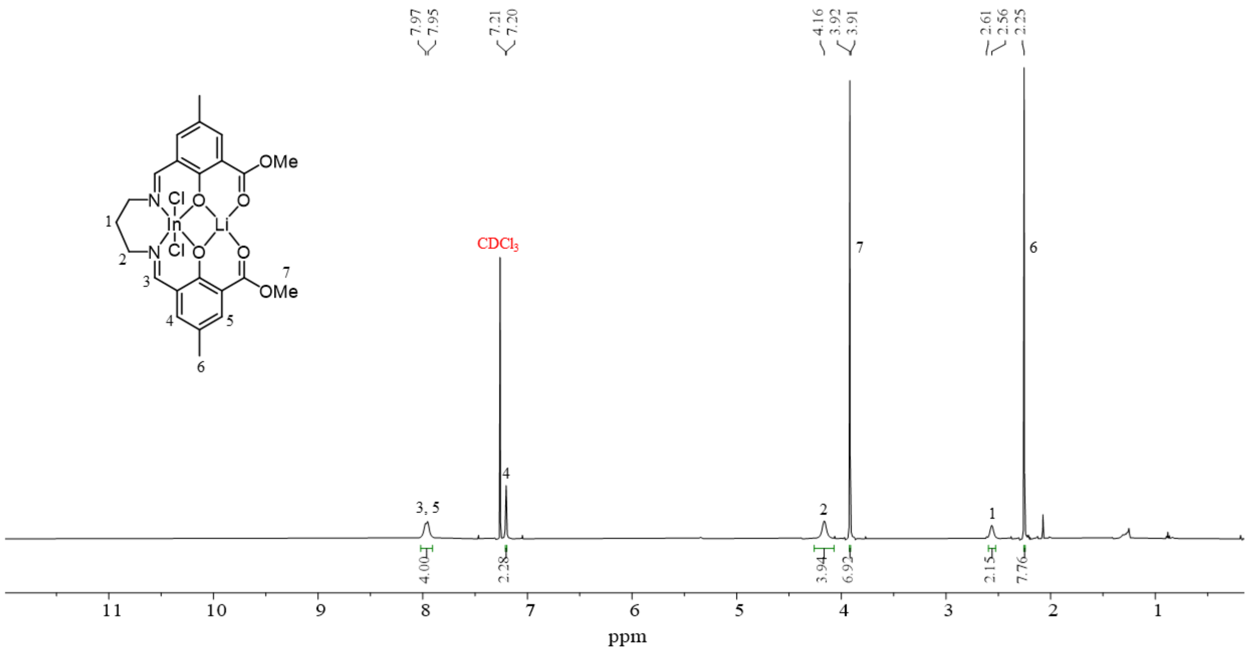

Figure S11. ^{1}H NMR spectrum (500 MHz, $CDCl_3$, 303K) of **$LLiInCl_2$**.

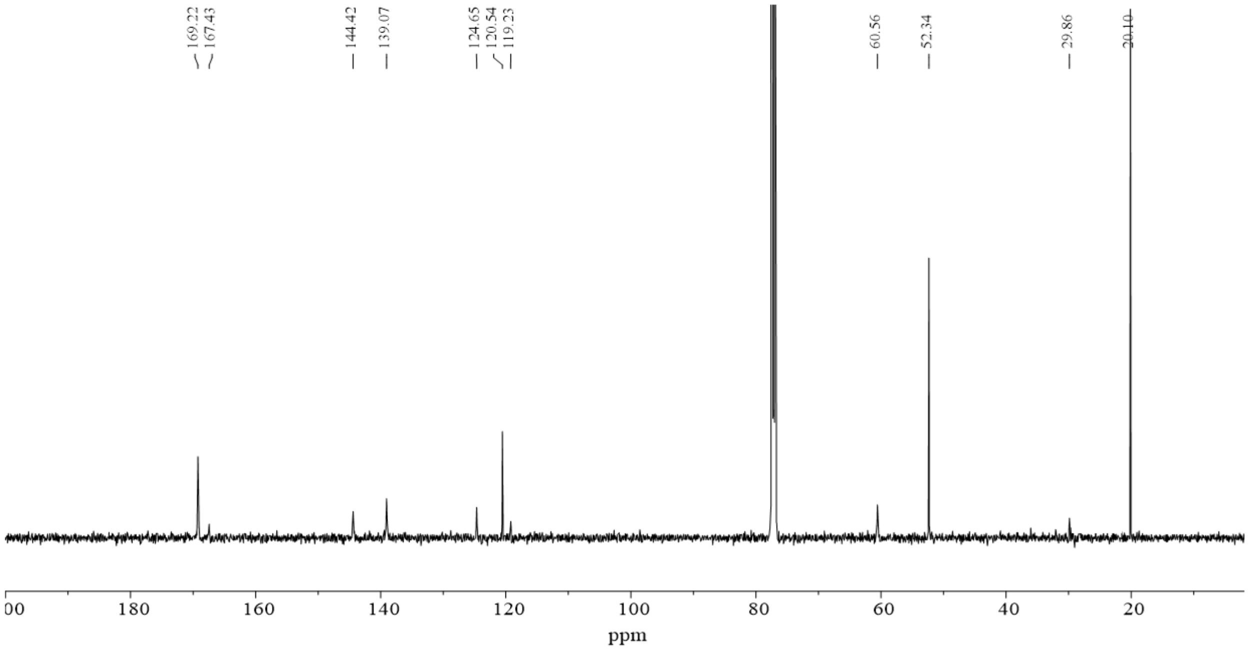

Figure S12. ^{13}C NMR spectrum (126 MHz, $CDCl_3$, 303K) of **$LLiInCl_2$.**

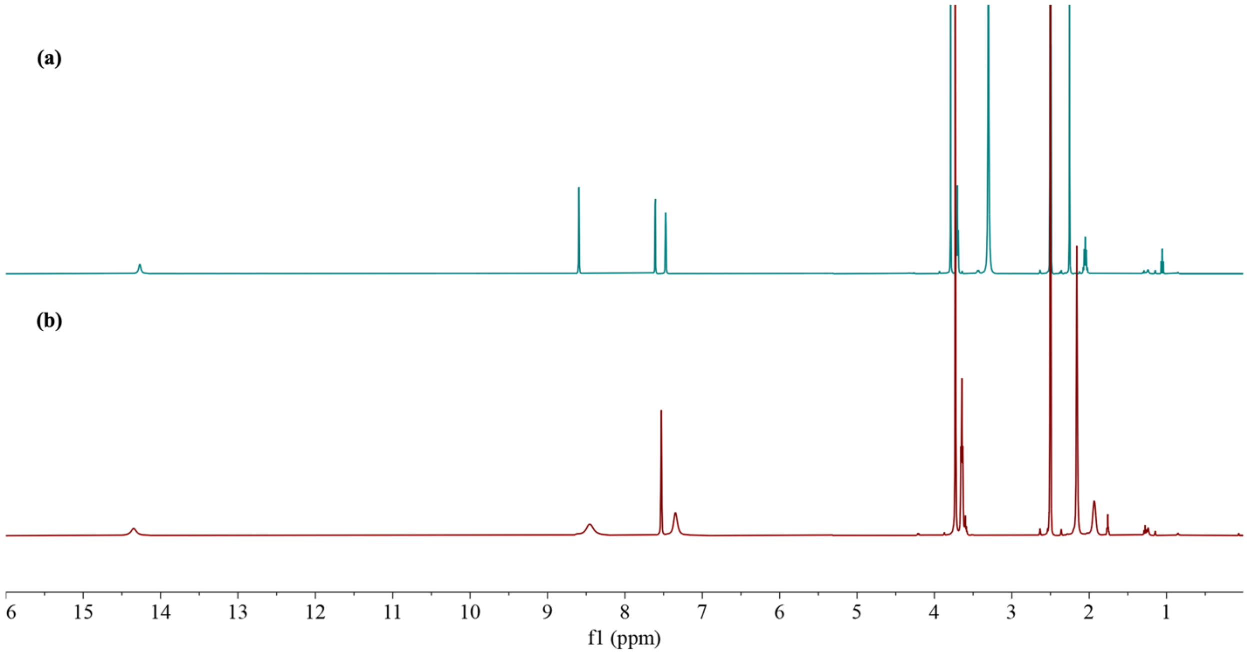

Figure S13. Series of ^{1}H NMR spectra (500 MHz, DMSO-d_6, 303K) of (a) **H_2L** and (b) followed by adding 2 equiv. of NaH. This reaction was stirred at room temperature overnight in THF, then isolated under vacuum and dissolved in DMSO-d_6 prior to analysis.

SEC data from *rac*-LA ROP

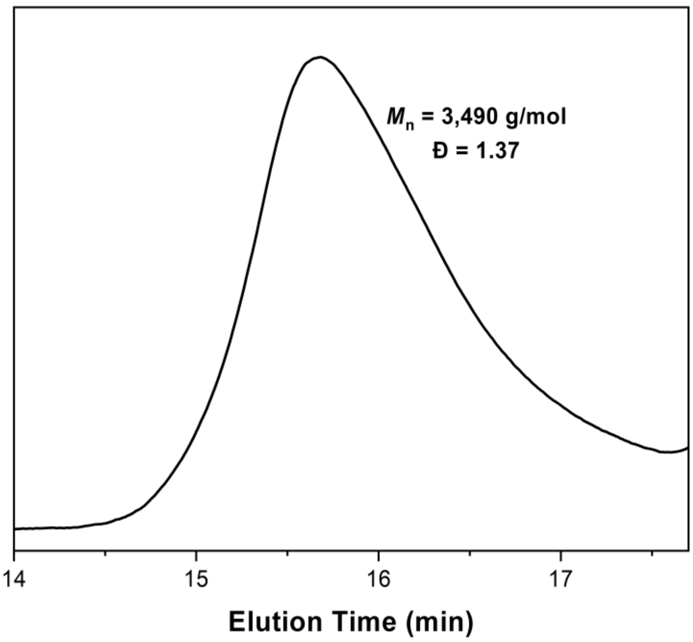

Figure S14. SEC trace of PLA catalysed by **LLiZnCl₂** (Table 1, entry 2).

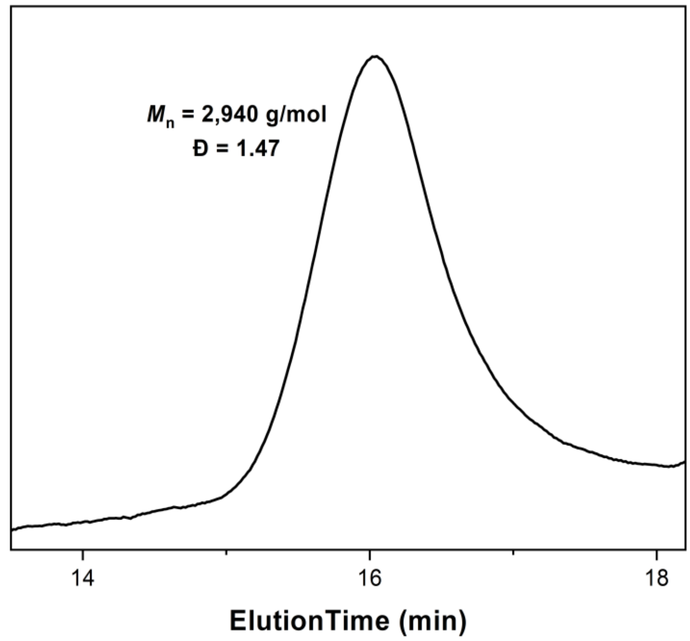

Figure S15. SEC trace of PLA catalysed by **LLiAlCl₂** (Table 1, entry 3).

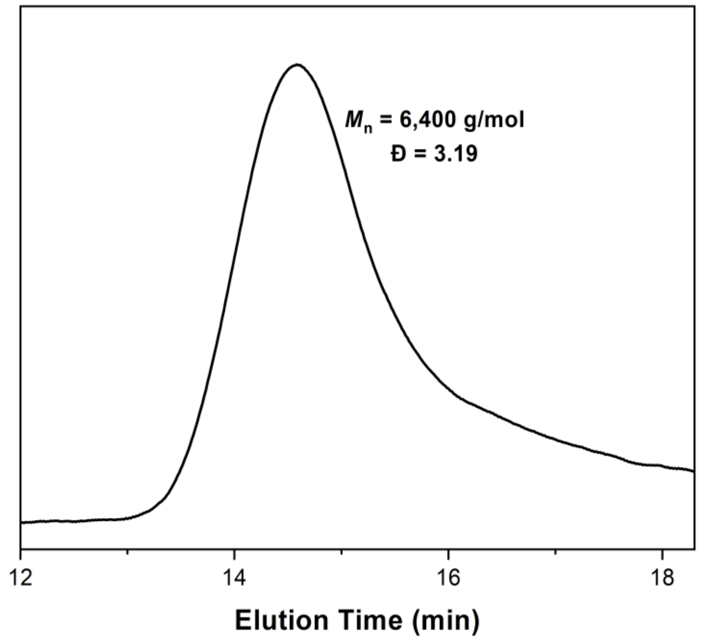

Figure S16. SEC traces of PLA catalysed by **LLiInCl₂** (Table 1, entry 4).

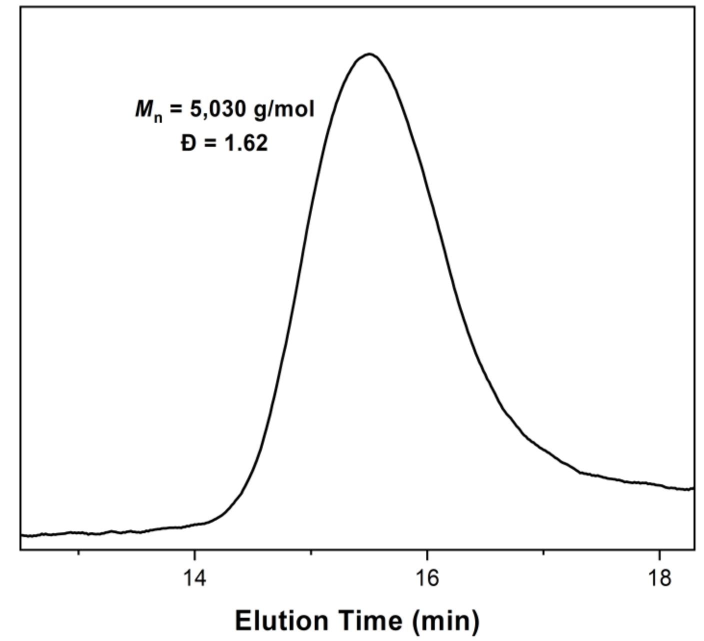

Figure S17. SEC traces of PLA catalysed by **LLiInCl₂** (Table 1, entry 5).

MALDI-ToF mass spectrometry of PLA

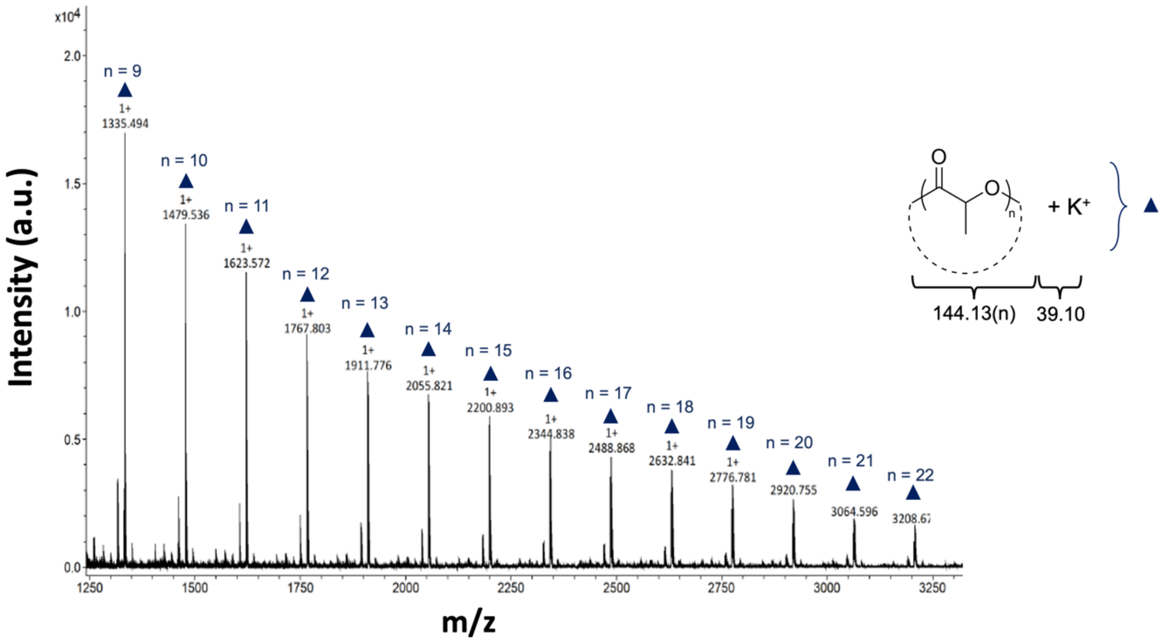

Figure S18. MALDI-ToF mass spectrum of PLA produced by **LLiZnCl** using PO as initiator.

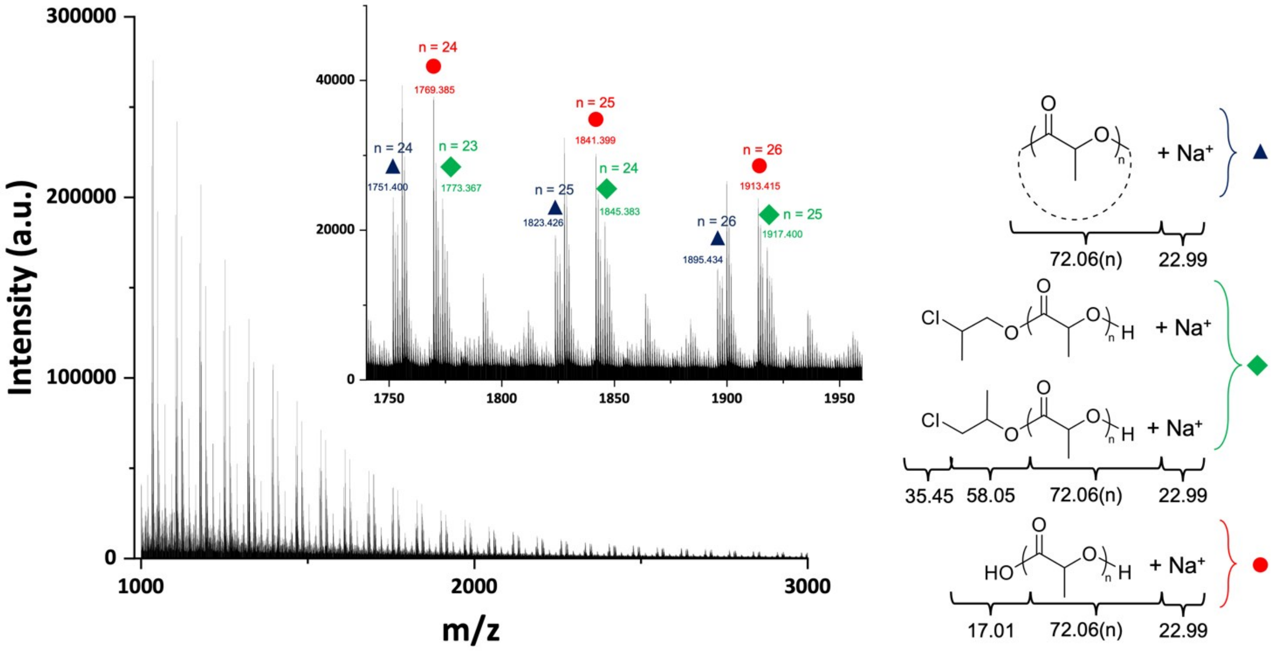

Figure S19. MALDI-ToF mass spectrum of PLA produced by **LLiAlCl₂** using PO as initiator.

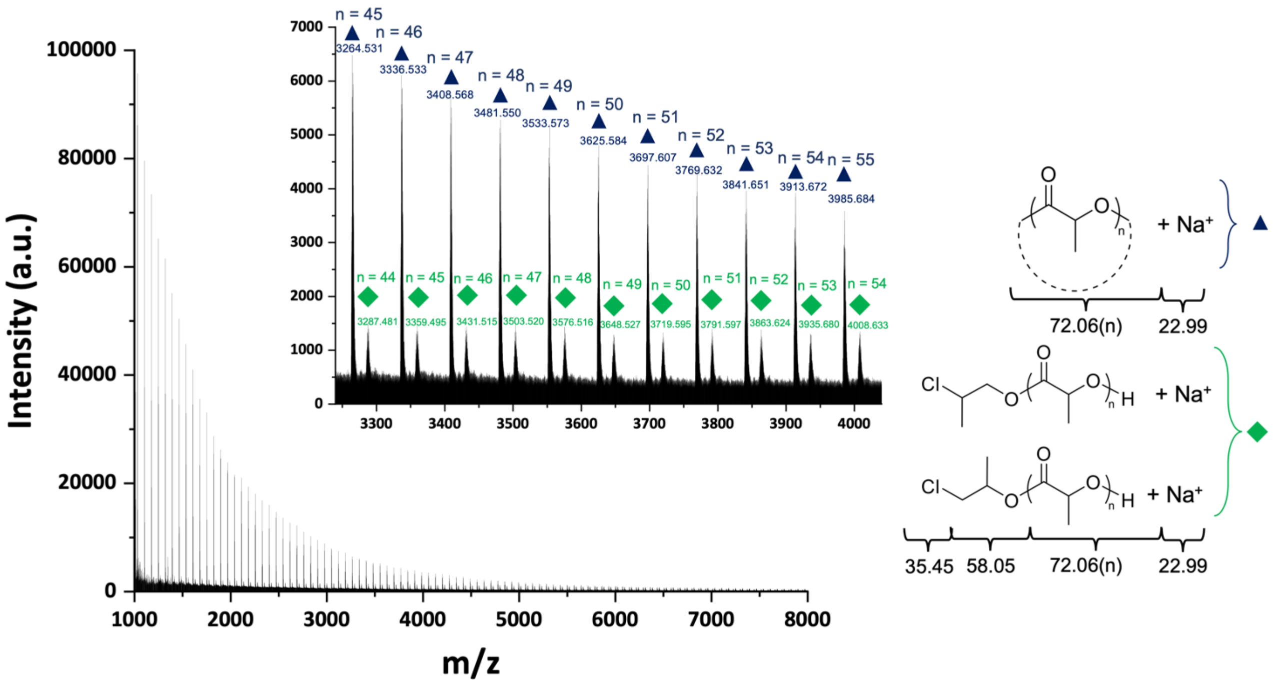

Figure S20. MALDI-ToF mass spectrum of PLA produced by **LLiInCl2** using PO as initiator.

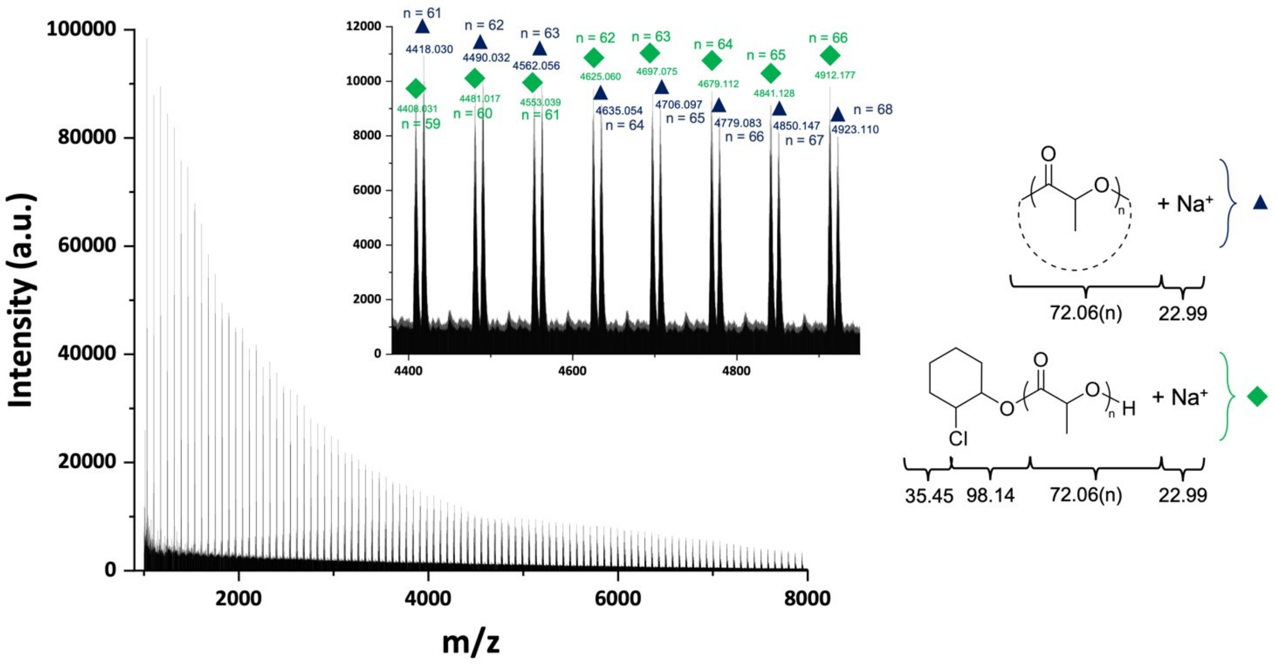

Figure S21. MALDI-ToF mass spectrum of PLA produced by **LLiInCl2** using CHO as initiator.

Example calculation of PLA tacticity

PLA tacticity was determined from the ^{1}H NMR spectrum (Figures S22–S24). The spectra were deconvoluted using MestReNova. The P_m values was calculated from each of tetrad integrals using Bernoulian statistical equations as shown below: [4]

$$[sis] = \frac{P_r^2}{2}$$

$$[sii] = \frac{P_r P_m}{2}$$

$$[iis] = \frac{P_r P_m}{2}$$

$$[iii] = P_m^2 + \frac{P_r P_m}{2}$$

$$[isi] = \frac{P_r^2 + P_r P_m}{2}$$

Tetrad	Integration	P_m
sis	0.11	0.52
sii	0.12	0.48
iis	0.12	0.48
iii	0.37	0.50
isi	0.27	0.45
**Average**		0.48

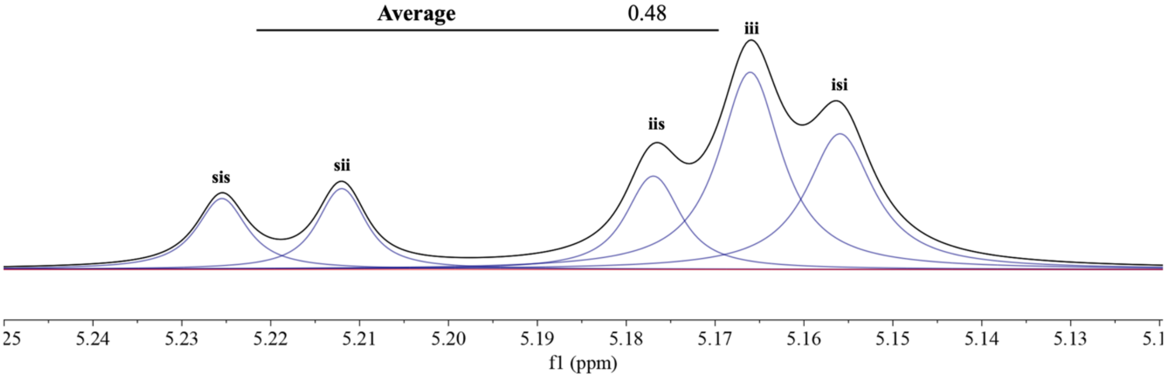

Figure S22. Pureshift-decoupled ^{1}H NMR spectrum (500 MHz, $CDCl_3$, 303K) of the methine region of PLA catalysed by **$LLiAlCl_2$** (Table 1, entry 3).

Tetrad	Integration	P_m
sis	0.19	0.39
sii	0.13	0.50
iis	0.14	0.54
iii	0.27	0.38
isi	0.28	0.47
**Average**		**0.45**

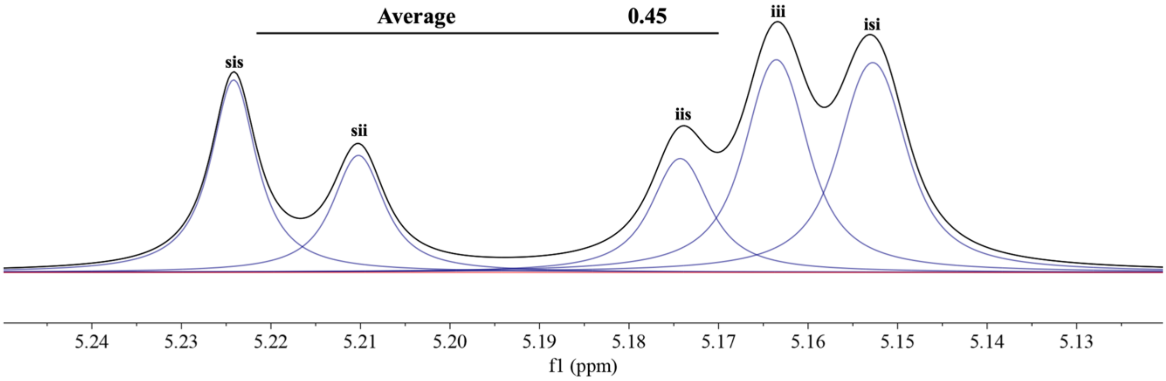

Figure S23. Pureshift-decoupled ^{1}H NMR spectrum (500 MHz, $CDCl_3$, 303K) of the methine region of PLA catalysed by **LLiInCl₂** (Table 1, entry 4).

Tetrad	Integration	P_m
sis	0.13	0.49
sii	0.12	0.48
iis	0.12	0.48
iii	0.30	0.42
isi	0.33	0.36
**Average**		**0.45**

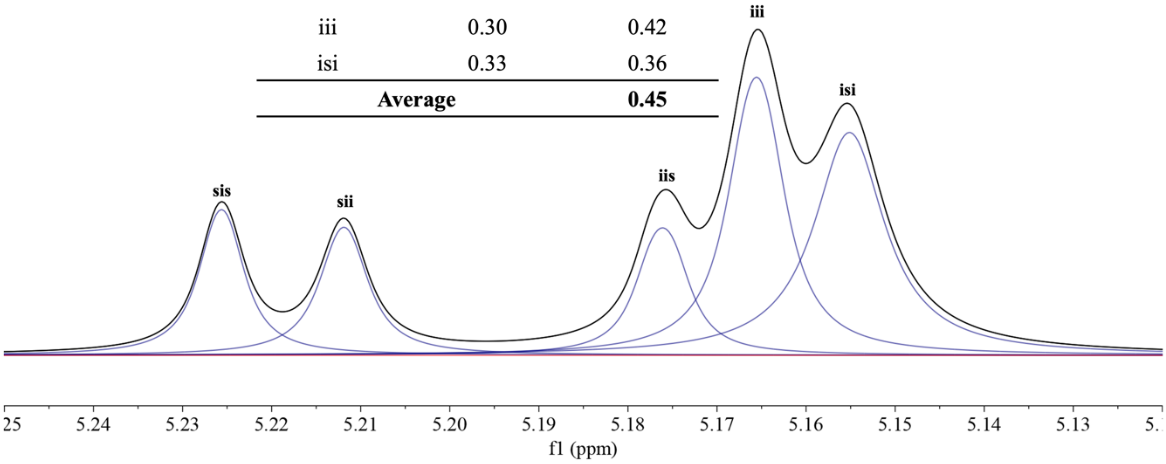

Figure S24. Pure shift ^{1}H NMR spectrum (500 MHz, $CDCl_3$, 303K) of the methine region of PLA catalysed by **LLiInCl₂** (Table 1, entry 5).

Example of purified PLA

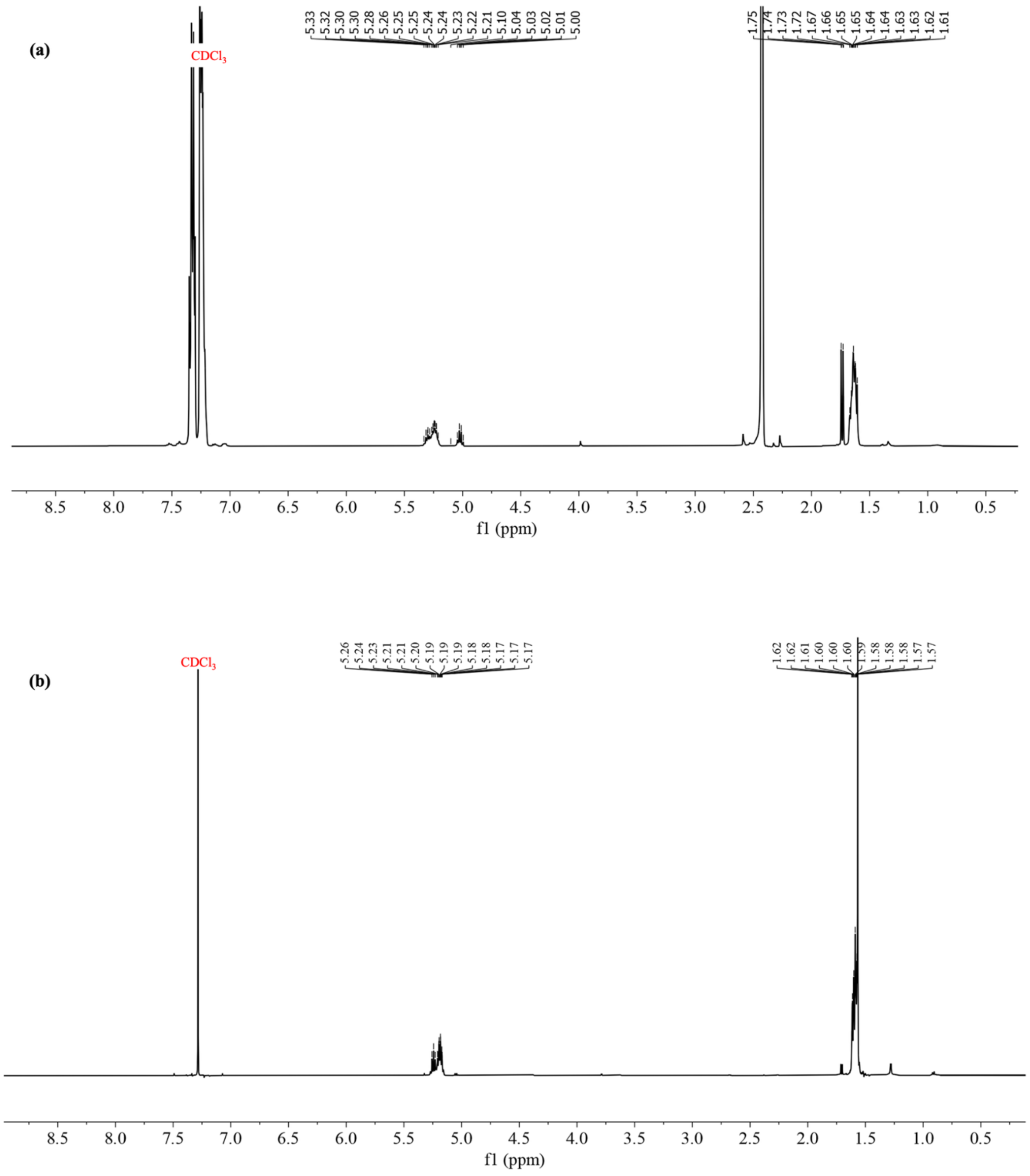

Figure S25. ^{1}H NMR spectrum (500 MHz, $CDCl_3$, 303K) of (a) crude mixture, and (b) purified PLA catalysed by **LLiInCl₂** from Table 1, entry 4. The absence of poly(propylene oxide) resonances in the region of 3.46–3.64 ppm, in both crude and purified polymer, indicates that PO is not homopolymerised by **LLiInCl₂**.

X-ray crystallography data

Table S1. Crystallographic data for **LLi_2**, **LLiZnCl**, **$LLiAlCl_2$**, and **$LLiInCl_2$**

Complex	LLi_2	LLiZnCl	$LLiAlCl_2$	$LLiInCl_2$
Formula	$C_{97}H_{106}Cl_{10}Li_8N_8O_{24}$	$C_{50.44}H_{56.88}Cl_{10.88}Li_2N_4O_{12}Zn_2$	$C_{25}H_{30}AlCl_4LiN_2O_7$	$C_{23}H_{24}Cl_2InLiN_2O_6$
$D_{calc.}$/ g cm^{-3}	1.389	1.520	1.478	1.638
μ/mm^{-1}	3.070	1.282	4.398	9.865
Formula Weight	2178.00	1441.47	646.23	617.120
Colour	colourless	pale green	clear colourless	clear colourless
Shape	needle	prism	lath-shaped	rod-shaped
Size/mm^3	0.24×0.08×0.06	0.46×0.19×0.15	0.11×0.04×0.02	0.20×0.07×0.05
T/K	120.00(10)	120.0	100.01(10)	100.00(10)
Crystal System	monoclinic	monoclinic	triclinic	monoclinic
Space Group	$P2_1/n$	$P2_1/c$	P-1	$P2_1/c$
a/Å	22.8774(4)	15.9150(3)	14.0391(2)	9.2410(1)
b/Å	19.3305(2)	24.9116(4)	14.1908(2)	19.9078(1)
c/Å	26.1585(4)	16.1264(3)	16.3182(4)	13.6849(1)
α/°	90	90	75.8345(18)	90
β/°	115.824(2)	99.907(2)	72.468(2)	96.153(1)
γ/°	90	90	71.7946(15)	90
V/Å^3	10412.9(3)	6298.3(2)	2903.82(11)	2503.08(4)
Z	4	4	4	4
Z'	1	1	2	1
Wavelength/Å	1.54184	0.71073	1.54184	1.54184
Radiation type	Cu K$_\alpha$	Mo K$_\alpha$	Cu K$_\alpha$	Cu K$_\alpha$
Θ_{min}/°	3.754	3.322	2.880	3.94
Θ_{max}/°	76.416	28.280	77.555	77.17
Measured Refl's.	210539	136091	75069	87855
Indep't Refl's	22026	15347	12077	5281
Refl's I≥2 σ(I)	18666	12382	10576	5042
R_{int}	0.1241	0.0594	0.0476	0.0397
Parameters	1403	785	752	532
Restraints	142	3	3	27
Largest Peak	2.464	2.381	0.989	1.3598
Deepest Hole	-1.266	-1.730	-0.752	-0.6364
GooF	1.023	1.021	1.054	1.0525
wR_2 (all data)	0.2514	0.1500	0.1046	0.0533
wR_2	0.2358	0.1428	0.1014	0.0528
R_1 (all data)	0.0998	0.0722	0.0449	0.0216
R_1	0.0872	0.0564	0.0390	0.0206

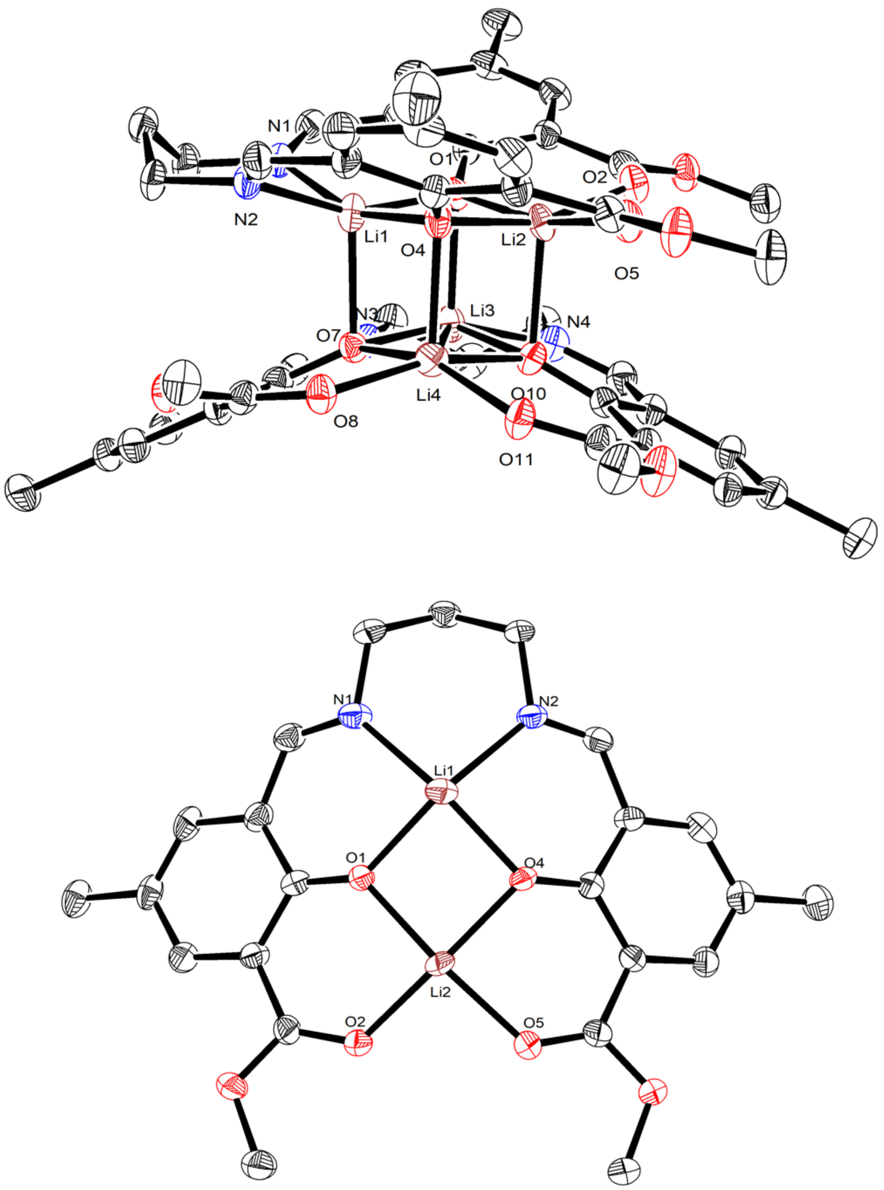

Figure S26. Dimeric (top) and monomeric (down) of molecular structure of **LLi_2** with displacement ellipsoids at the 50% probability level and hydrogen atoms removed for clarity.

Due to the similarity of bond lengths and angles only one set of the equivalent atoms are reported.

Table S2. Selected bond distances in Å for complex **LLi_2**

Atom	Atom	Length/ Å
O1	Li1	1.986(8)
O1	Li2	1.987(8)
O1	Li3	2.100(8)
O2	Li2	1.971(8)
O4	Li1	2.010(9)
O4	Li2	2.004(8)
O4	Li4	2.075(8)
O5	Li2	2.016(8)
O7	Li1	2.089(9)

Atom	Atom	Length/ Å
O7	Li3	1.976(8)
O7	Li4	1.987(8)
O8	Li4	2.009(8)
O10	Li2	2.105(8)
O10	Li3	2.020(8)
O10	Li4	2.005(8)
O11	Li4	2.024(8)
N1	Li1	2.112(9)
N2	Li1	2.130(8)
N3	Li3	2.152(8)
N4	Li3	2.146(8)
Li1	Li2	2.729(1)

Table S3. Selected bond angles in ° for complex **LLi_2**

Atom	Atom	Atom	Angle/°
Li1	O1	Li2	93.6(3)
Li1	O1	Li3	86.6(3)
Li2	O1	Li3	87.3(3)
Li1	O4	Li4	85.7(3)
Li2	O4	Li1	92.3(3)
Li2	O4	Li4	86.4(3)
Li3	O7	Li1	87.1(3)
Li3	O7	Li4	93.0(3)
Li4	O7	Li1	85.9(3)
Li3	O10	Li2	86.3(3)
Li4	O10	Li2	85.6(3)
Li4	O10	Li3	91.2(3)
O1	Li1	O4	86.1(3)
O1	Li1	O7	93.1(3)
O1	Li1	N1	87.7(3)
O1	Li1	N2	157.4(5)
O4	Li1	O7	93.5(3)
O4	Li1	N1	160.0(5)
O4	Li1	N2	87.4(3)
O7	Li1	N1	105.8(4)
O7	Li1	N2	109.0(4)
N1	Li1	N2	91.0(3)
O1	Li2	O4	86.3(3)
O1	Li2	O5	165.1(5)
O1	Li2	O10	93.5(3)
O2	Li2	O1	89.4(3)

Atom	Atom	Atom	Angle/°
O2	Li2	O4	161.8(5)
O2	Li2	O5	91.9(3)
O2	Li2	O10	104.4(4)
O4	Li2	O5	87.9(3)
O4	Li2	O10	93.5(3)
O5	Li2	O10	100.5(4)
O1	Li3	N3	109.6(4)
O1	Li3	N4	105.3(3)
O7	Li3	O1	93.0(3)
O7	Li3	O10	86.9(3)
O7	Li3	N3	87.9(3)
O7	Li3	N4	161.0(4)
O10	Li3	O1	92.7(3)
O10	Li3	N3	157.3(4)
O10	Li3	N4	87.1(3)
N4	Li3	N3	90.6(3)
O7	Li4	O4	94.6(3)
O7	Li4	O8	89.0(3)
O7	Li4	O10	87.0(3)
O7	Li4	O11	159.5(4)
O8	Li4	O4	100.3(3)
O8	Li4	O11	91.8(3)
O10	Li4	O4	94.4(3)
O10	Li4	O8	165.1(4)
O10	Li4	O11	87.0(3)
O11	Li4	O4	105.3(4)

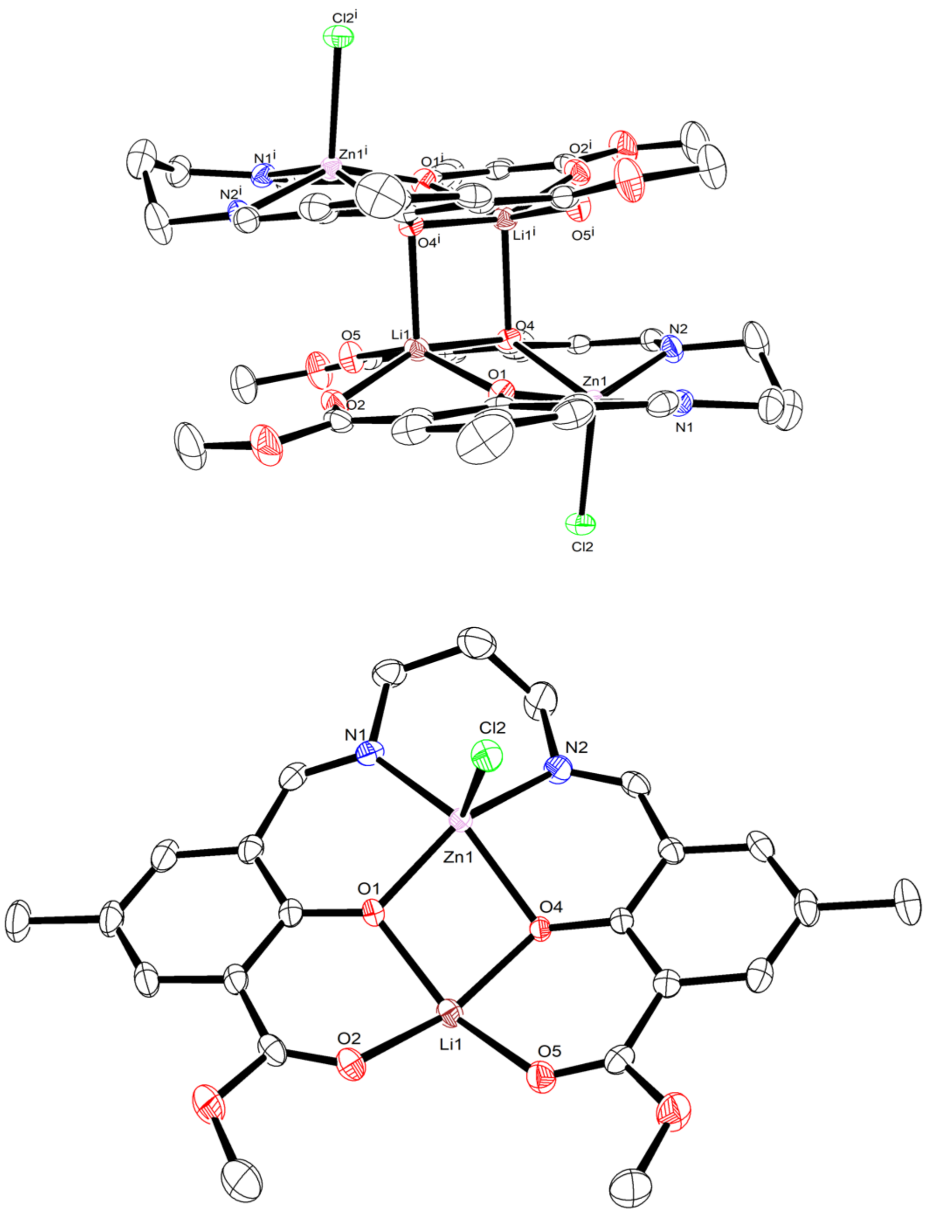

Figure S27. Dimeric (top) and monomeric (down) of molecular structure of **LLiZnCl** with displacement ellipsoids at the 50% probability level and hydrogen atoms removed for clarity.

Due to the similarity of bond lengths and angles only one set of the equivalent atoms are reported.

Table S4. Selected bond distances in Å for complex **LLiZnCl**

**Atom**	**Atom**	**Length/ Å**
Zn1	Cl2	2.299(8)
Zn1	O1	2.007(2)
Zn1	O4	2.108(2)
Zn1	N1	2.088(3)
Zn1	N2	2.090(3)
O1	Li1	1.982(6)
O2	Li1	1.972(6)
O4	Li1	2.001(6)
O4	Li1[1]	2.112(6)
O5	Li1	1.965(6)
Li1	Li1[1]	2.744(1)
Zn1	Li1	3.067(1)

[1]1-x,1-y,2-z

Table S5. Selected bond angles in ° for complex **LLiZnCl**

**Atom**	**Atom**	**Atom**	**Angle/°**
O1	Zn1	Cl2	104.9(7)
O1	Zn1	O4	79.2(8)
O1	Zn1	N1	88.9(1)
O1	Zn1	N2	151.0(1)
O4	Zn1	Cl2	100.8(6)
N1	Zn1	Cl2	110.8(8)
N1	Zn1	O4	148.2(9)
N1	Zn1	N2	92.8(1)
N2	Zn1	Cl2	101.5(8)
N2	Zn1	O4	84.3(1)
Li1	O1	Zn1	100.5(2)
Zn1	O4	Li1[1]	110.7(2)
Li1	O4	Zn1	96.5(2)
Li1	O4	Li1[1]	83.6(2)
O1	Li1	O4	82.5(2)
O1	Li1	O4[1]	102.3(2)
O1	Li1	Li1[1]	93.9(3)
O2	Li1	O1	86.1(2)
O2	Li1	O4	158.3(3)
O2	Li1	O4[1]	104.0(2)
O2	Li1	Li1[1]	149.8(4)
O4	Li1	O4[1]	96.4(2)
O4	Li1	Li1[1]	49.9(2)
O4[1]	Li1	Li1[1]	46.5(2)
O5	Li1	O1	151.7(3)
O5	Li1	O2	93.6(2)
O5	Li1	O4[1]	105.2(3)
O5	Li1	O4	88.0(2)
O5	Li1	Li1[1]	100.1(3)

[1]1-x,1-y,2-z

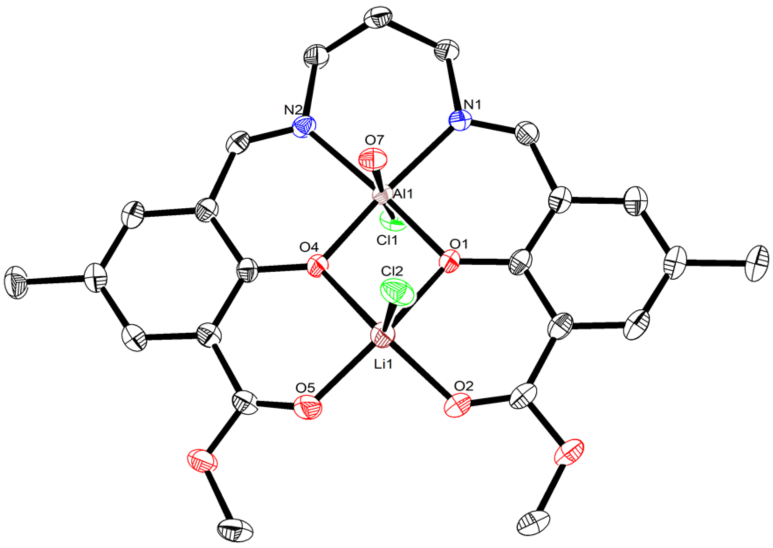

Figure S28. The molecular structure of **LLiAlCl$_2$·H$_2$O** with displacement ellipsoids at the 50% probability level and hydrogen atoms removed for clarity.

Table S6. Selected bond distances in Å for complex **LLiAlCl$_2$·H$_2$O**

Atom	Atom	Length/ Å
Cl1	Al1	2.279(7)
Cl2	Li1	2.508(4)
Al1	O1	1.853(1)
Al1	O4	1.862(1)
Al1	O7	1.957(2)
Al1	N1	2.047(2)
Al1	N2	2.023(2)
O1	Li1	2.016(4)
O2	Li1	1.901(4)
O4	Li1	1.998(4)
O5	Li1	1.905(4)
Al1	Li1	2.948(1)

Table S7. Selected bond angles in ° for complex **LLiAlCl$_2$·H$_2$O**

Atom	Atom	Atom	Angle/°
O1	Al1	Cl1	94.8(5)
O1	Al1	O4	83.2(6)
O1	Al1	O7	89.2(6)
O1	Al1	N1	90.9(6)
O1	Al1	N2	173.0(7)
O4	Al1	Cl1	98.0(5)
O4	Al1	O7	86.7(7)
O4	Al1	N1	171.8(7)
O4	Al1	N2	91.5(6)
O7	Al1	Cl1	174.2(5)
O7	Al1	N1	87.6(7)
O7	Al1	N2	86.0(7)
N1	Al1	Cl1	88.1(5)

Atom	Atom	Atom	Angle/°
N2	Al1	N1	94.0(7)
Al1	O1	Li1	99.2(1)
Al1	O4	Li1	99.5(1)
O1	Li1	Cl2	96.6(2)
O2	Li1	Cl2	104.2(2)
O2	Li1	O1	88.1(2)
O2	Li1	O4	145.2(2)
O2	Li1	O5	92.8(2)
O4	Li1	Cl2	108.1(2)
O4	Li1	O1	75.8(1)
O5	Li1	Cl2	106.2(2)
O5	Li1	O1	156.2(2)
O5	Li1	O4	90.4(2)

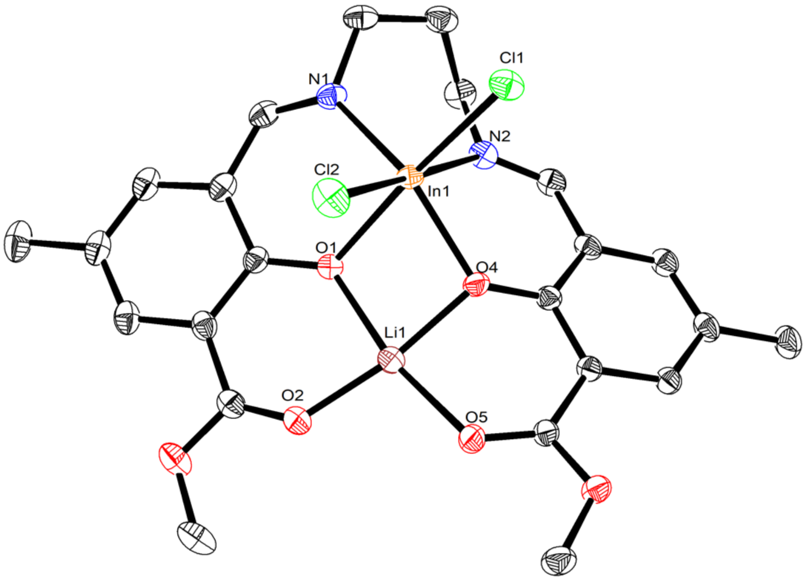

Figure S29. The molecular structure of **$LLiInCl_2$** with displacement ellipsoids at the 50% probability level and hydrogen atoms removed for clarity.

Table S8. Selected bond distances in Å for complex **$LLiInCl_2$**

Atom	Atom	Length/ Å
In1	Cl1	2.448(4)
In1	Cl2	2.465(5)
In1	O1	2.189(1)
In1	O4	2.116(1)
In1	N1	2.207(2)
In1	N2	2.306(2)
O1	Li1	2.035(4)
O2	Li1	1.882(4)
O4	Li1	1.908(3)
O5	Li1	2.003(4)
In1	Li1	3.079(1)

Table S9. Selected bond angles in ° for complex **$LLiInCl_2$**

Atom	Atom	Atom	Angle/°
Cl2	In1	Cl1	91.0(2)
O1	In1	Cl1	171.9(3)
O1	In1	Cl2	83.4(4)
O4	In1	Cl1	101.1(4)
O4	In1	Cl2	99.8(4)
O4	In1	O1	74.1(5)
N1	In1	Cl1	103.5(4)
N1	In1	Cl2	100.7(4)
N1	In1	O1	83.5(5)
N1	In1	O4	147.4(5)
N2	In1	Cl1	85.4(4)
N2	In1	Cl2	176.5(4)

Atom	Atom	Atom	Angle/°
N2	In1	O1	100.0(5)
N2	In1	O4	80.5(5)
N2	In1	N1	80.6(6)
Li1	O1	In1	93.6(1)
Li1	O4	In1	99.7(1)
O2	Li1	O1	88.0(1)
O4	Li1	O1	82.2(1)
O4	Li1	O2	170.2(2)
O5	Li1	O1	168.4(2)
O5	Li1	O2	103.6(2)
O5	Li1	O4	86.2(1)

Computational methods

Ab initio molecular orbital calculations: A series of geometry optimisation calculations were performed for **LM1M2Cl** (where M1 = the inner pocket metal (Zn, In) and M2 = the outer pocket metal (Li)) complexes using Gaussian 16 and utilising the B3LYP/6-311G* model chemistry.[5] Models were built from the X-ray coordinates of related system(s), edited with Z-matrix. All optimised stationary points were confirmed as minima by vibrational frequency analysis (all real numbers) and recast as free energies following computation of thermochemical energy corrections. Local force constant analysis was performed to extract the force constants for the specified bond distances from the computed normal mode vibrational frequencies using LModeA-NANO.[6]

Table S10. Free Gibbs energy for different epoxide/chloride(s) coordination on the metal centres of **LLiZnCl**

M1 (Inner)	M2 (Outer)	Chloride/epoxide coordination position	Free energy (Hartrees)	ΔG (kJ mol^{-1})
Zn	Li	epoxide on **Li**, on same face as Cl on Zn	-3852.873697	0
		epoxide on **Li**, on opposite face as Cl on Zn	-3852.868546	+13.5
		epoxide on **Zn**, on opposite face as Cl on Li	-3852.867852	+15.3

Table S11. Comparison of Zn—Cl bond lengths and force constants of **LLiZnCl** before and upon epoxide coordination.

Bond	Epoxide coordination	Bond length (Å)	Force constant (mdyn/Å)
Zn—Cl	Before	2.303	0.958
	After	2.330	0.851

Table S12. Free Gibbs energy for different epoxide/chloride(s) coordination on the metal centres of **LLiInCl$_2$**

M1 (Inner)	M2 (Outer)	Chloride/epoxide coordination position	Free energy (Hartrees)	ΔG (kJ mol^{-1})
In	Li	epoxide on **Li**, on same face as Cl on In	-3852.873697	0
		epoxide on **Li**, on opposite face as Cl on In	-3852.868546	+4.0

Note: Epoxide bonding to In was investigated but not observed.

Table S13. Comparison of In—Cl bond lengths and force constants of **LLiInCl$_2$** before and upon epoxide coordination.

Bond	Epoxide coordination	Bond length (Å)	Force constant (mdyn/Å)
In—Cl	Before	2.427/2.489	1.147/0.78
	After	2.441/2.449	1.078/1.037

Table S14. Comparison of force constants of **LLiInCl₂** before and upon epoxide coordination.

	Local force constants (m dyn Å^{-1})		
**Bond type**	**LiIn no epoxide**	**LiIn epoxide on Li, opposite face to In-Cl**	**LiIn epoxide on Li, same face as In-Cl**
In-N	1.012/0.718	1.012/0.509	1.075/0.698
In-O	1.263/0.752	1.291/0.733	1.207/0.868
In-Cl	1.147/0.78	1.149/1.05	1.078/1.037
Li-O	0.522/0.44/0.382/0.075	0.589/0.501/0.285/0.16	0.451/0.32/0.303/0.186
Li-$O_{epoxide}$	-	0.232	0.25

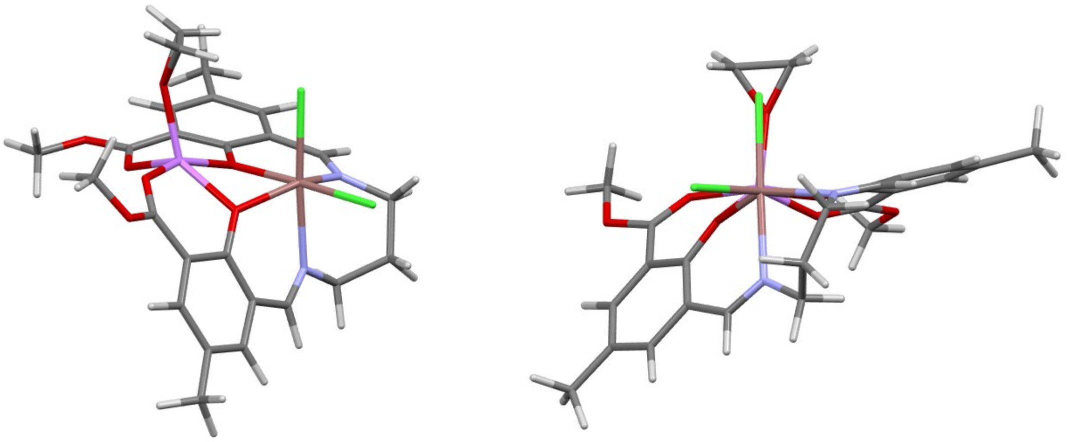

Figure S30. Top (left) and side (right) views of geometry optimised complex **LLiInCl₂** of the more stable configurations upon epoxide coordination. Colour scheme: grey = C, light purple = N, red = O, dark purple = Zn, green = Cl, pink = Li.

Overview of heterometallic systems reported in literature

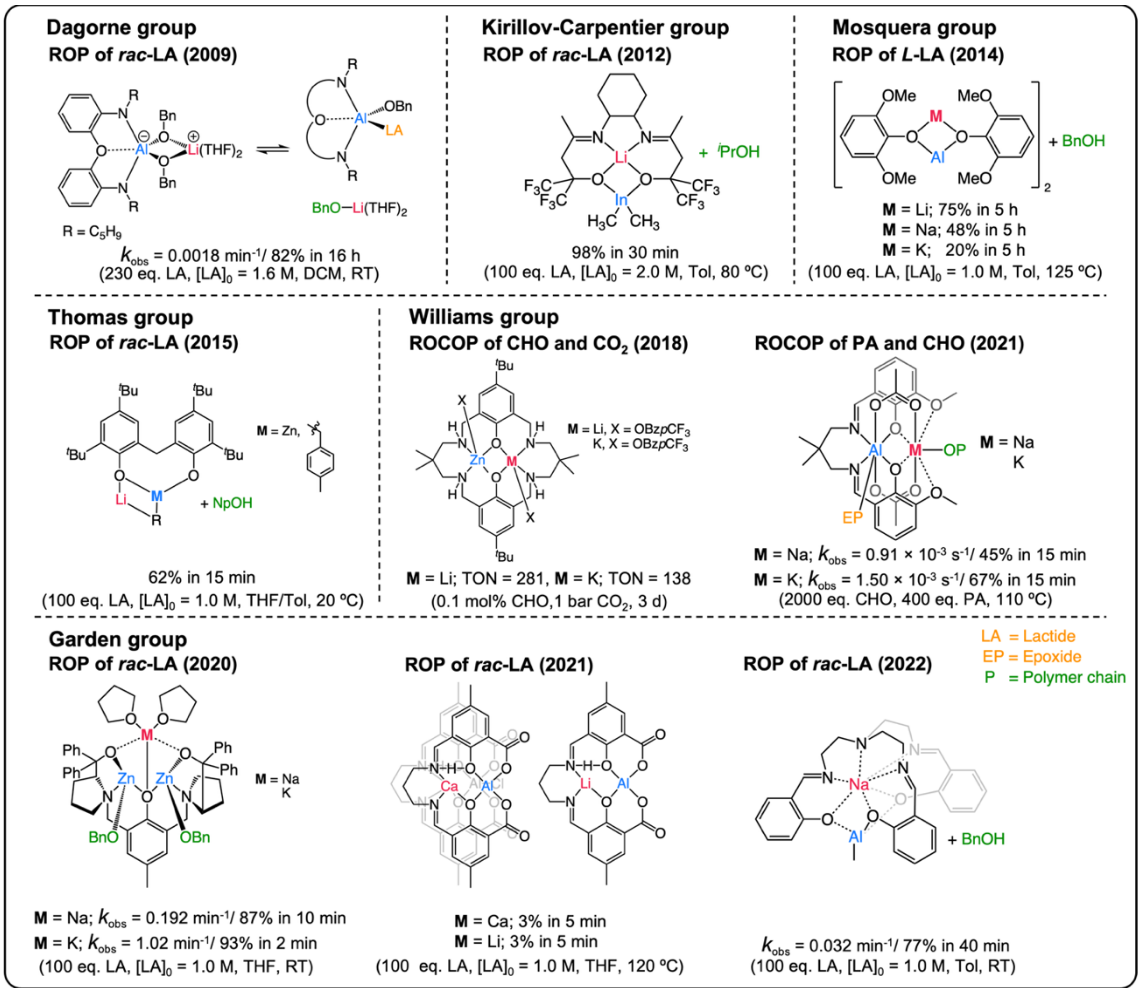

Figure S31. Overview of heterometallic systems reported in literature that combine an s block metal with Zn, Al, or In for RO(CO)P.[7-15]

The features of heterometallic "ate" catalysts

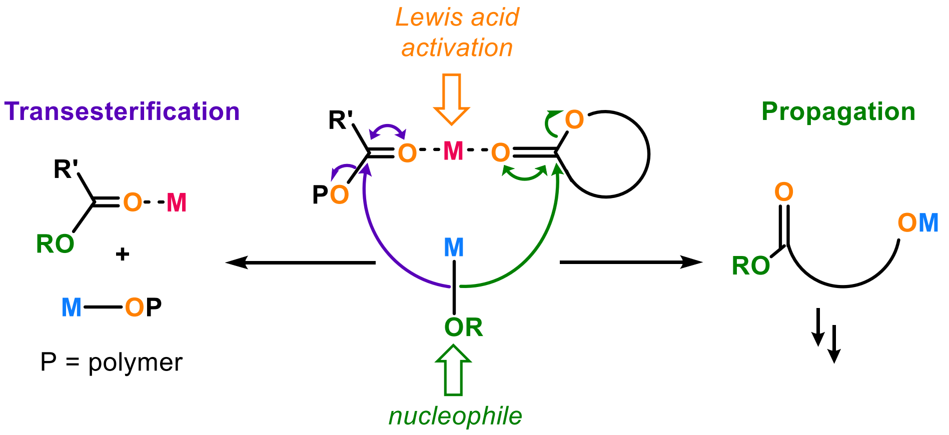

Figure S32. The features of heterometallic "ate" catalysts that enhance propagation can also enhance transesterification.[16]

References

1. N. Krogsgaard-Larsen, C. G. Delgar, K. Koch, P. M. G. E. Brown, C. Møller, L. Han, T. H. V. Huynh, S. W. Hansen, B. Nielsen, D. Bowie, D. S. Pickering, J. S. Kastrup, K. Frydenvang and L. Bunch, Design and synthesis of a series of l-trans-4-substituted srolines as selective antagonists for the ionotropic glutamate receptors including functional and X-ray crystallographic studies of new subtype selective kainic acid receptor subtype 1 (GluK1) antagonist (2S,4R)-4-(2-carboxyphenoxy)pyrrolidine-2-carboxylic acid, *J. Med. Chem.*, 2017, **60**, 441-457.
2. G. Sheldrick, Crystal structure refinement with SHELXL, *Acta Crystallogr. Sect. C*, 2015, **71**, 3-8.
3. O. V. Dolomanov, L. J. Bourhis, R. J. Gildea, J. A. K. Howard and H. Puschmann, OLEX2: a complete structure solution, refinement and analysis program, *J. Appl. Crystallogr.*, 2009, **42**, 339-341.
4. B. M. Chamberlain, M. Cheng, D. R. Moore, T. M. Ovitt, E. B. Lobkovsky and G. W. Coates, Polymerization of lactide with zinc and magnesium β-diiminate complexes: stereocontrol and mechanism, *J. Am. Chem. Soc.*, 2001, **123**, 3229-3238.
5. M. J. Frisch, G. W. Trucks, H. B. Schlegel, G. E. Scuseria, M. A. Robb, J. R. Cheeseman, G. Scalmani, V. Barone, G. A. Petersson, H. Nakatsuji, X. Li, M. Caricato, A. V. Marenich, J. Bloino, B. G. Janesko, R. Gomperts, B. Mennucci, H. P. Hratchian, J. V. Ortiz, A. F. Izmaylov, J. L. Sonnenberg, Williams, F. Ding, F. Lipparini, F. Egidi, J. Goings, B. Peng, A. Petrone, T. Henderson, D. Ranasinghe, V. G. Zakrzewski, J. Gao, N. Rega, G. Zheng, W. Liang, M. Hada, M. Ehara, K. Toyota, R. Fukuda, J. Hasegawa, M. Ishida, T. Nakajima, Y. Honda, O. Kitao, H. Nakai, T. Vreven, K. Throssell, J. A. Montgomery Jr., J. E. Peralta, F. Ogliaro, M. J. Bearpark, J. J. Heyd, E. N. Brothers, K. N. Kudin, V. N. Staroverov, T. A. Keith, R. Kobayashi, J. Normand, K. Raghavachari, A. P. Rendell, J. C. Burant, S. S. Iyengar, J. Tomasi, M. Cossi, J. M. Millam, M. Klene, C. Adamo, R. Cammi, J. W. Ochterski, R. L. Martin, K. Morokuma, O. Farkas, J. B. Foresman and D. J. Fox, Gaussian 16 Rev. C.01.*Journal*, 2016.
6. E. Kraka, W. Zou and Y. Tao, Decoding chemical information from vibrational spectroscopy data: Local vibrational mode theory, *WIREs Comput. Mol. Sci.*, 2020, **10**, e1480.
7. F. Hild, P. Haquette, L. Brelot and S. Dagorne, Synthesis and structural characterization of well-defined anionic aluminium alkoxide complexes supported by NON-type diamido ether tridentate ligands and their use for the controlled ROP of lactide, *Dalton Trans.*, 2010, **39**, 533-540.
8. M. Normand, E. Kirillov, T. Roisnel and J.-F. Carpentier, Indium complexes of fluorinated dialkoxy-diimino salen-like ligands for ring-opening polymerization of *rac*-lactide: how does indium compare to aluminum?, *Organometallics*, 2012, **31**, 1448-1457.
9. M. T. Muñoz, T. Cuenca and M. E. G. Mosquera, Heterometallic aluminates: alkali metals trapped by an aluminium aryloxide claw, *Dalton Trans.*, 2014, **43**, 14377-14385.
10. E. B. Joëlle Char, Philippe C. Gros, Marie-Noelle Rager, Vincent Guérineau, Christophe M. Thomas, Synthesis of heterotactic PLA from *rac*-lactide using hetero-bimetallic Mg/Zn–Li systems, *J. Organomet. Chem.*, 2015, **796**, 47-52.
11. A. C. Deacy, C. B. Durr, J. A. Garden, A. J. P. White and C. K. Williams, Groups 1, 2 and Zn(II) heterodinuclear catalysts for epoxide/CO_2 ring-opening copolymerization, *Inorg. Chem.*, 2018, **57**, 15575-15583.

12. W. T. Diment, G. L. Gregory, R. W. F. Kerr, A. Phanopoulos, A. Buchard and C. K. Williams, Catalytic synergy using Al(III) and group 1 metals to accelerate epoxide and anhydride ring-opening copolymerizations, *ACS Catal.*, 2021, **11**, 12532-12542.
13. W. Gruszka, A. Lykkeberg, G. S. Nichol, M. P. Shaver, A. Buchard and J. A. Garden, Combining alkali metals and zinc to harness heterometallic cooperativity in cyclic ester ring-opening polymerisation, *Chem. Sci.*, 2020, **11**, 11785-11790.
14. Y. Zhou, G. S. Nichol and J. A. Garden, Incorporating sodium to boost the activity of aluminium TrenSal complexes towards *rac*-lactide polymerisation, *Eur. J. Inorg. Chem.*, 2022, **2022**, e202200134.
15. A. J. Gaston, Z. Greindl, C. A. Morrison and J. A. Garden, Cooperative heterometallic catalysts for lactide ring-opening polymerization: combining aluminum with divalent metals, *Inorg. Chem.*, 2021, **60**, 2294-2303.
16. M. Abdul Rahman, T. J. Neal and J. A. Garden, Cooperative heterometallic catalysts: balancing activity and control in PCL-block-PLA copolymer synthesis, *Chem. Commun.*, 2024, **60**, 5530-5533.
